# Synthesis of a 3D chitosan/cellulose acetate hydrogel: comparative impact of drying techniques on morphology for sustainable wastewater treatment

**DOI:** 10.1038/s41598-026-53193-0

**Published:** 2026-06-08

**Authors:** Laila Mohamed, Wafaa K. Mekhamer, Hemmat A. Elbadawy, Doaa S. El-Sayed, Amel F. Elhussieny, Ali El-Dissouky

**Affiliations:** 1 Chemistry Department, Faculty of Science, Alexandria University, Alexandria, Egypt; 2 Department of Material Science, Institute of Graduate Studies & Research, Alexandria University, Alexandria, Egypt

**Keywords:** Biopolymer hydrogels, Freeze-drying, Dye capture, Computational study, Adsorption mechanism, Chemistry, Engineering, Environmental sciences, Materials science

## Abstract

Towards achieving the sustainable development goals, this work contributes to long-term improvements in water and sanitation infrastructure by developing new adsorbents from two naturally based polymers. To address this issue, a novel 3D chitosan/cellulose acetate hydrogel (CCA-HG), crosslinked with minimal glutaraldehyde, was synthesized and dried using two drying techniques: freeze-drying (CCA-HG_F_) and air-drying (CCA-HG_A_). The impact of the two drying techniques affected the adsorptive efficiency of the two products towards hazardous anionic textile dye. Both hydrogels were comprehensively characterized by FTIR, SEM, XPS, XRD, BET, and swelling capacity, confirming successful crosslinking and distinct morphologies. However, CCA-HG_F_ showed a softer, more porous texture with greater surface area and swelling capacity than CCA-HG_A_. Applying the batch method indicated that the freeze-drying method produced a better and more efficient adsorbent since CCA-HG_F_ achieved an adsorption capacity of 149.30 mg/g and 99.53% efficiency within 75 min, whereas CCA-HG_A_ demonstrated 20.50 mg/g, 98.44% in 90 min. Adsorption followed a pseudo-second-order model and reached equilibrium within 75–90 min. Isothermal and thermodynamic studies indicated a spontaneous, exothermic, hybrid physical-chemical process. Both hydrogels maintained excellent reusability over five adsorption-desorption cycles. Complementary computational calculations; DFT and molecular dynamics simulations revealed a reduced energy gap (4.055 to 2.476 eV), enhanced charge transfer, and strong non-covalent interactions (–24.544 to − 22.312 Kcal/mol), upon adsorption. To the best of our knowledge, this study establishes freeze-drying as a key strategy for fabricating highly porous, high-capacity biopolymer adsorbents, bridging a crucial gap between sustainable material synthesis and high-performance application.

**Supplementary Information:**

The online version contains supplementary material available at 10.1038/s41598-026-53193-0.

## Introduction

Environmental pollution is rising due to population growth and various human activities, threatening essential resources, especially water. Around 5 million people die annually from contaminated water. Wastewater treatment is crucial for conserving water resources, as it contains pollutants like heavy metals, fertilizers, drugs, and synthetic dyes, which harm human health and ecosystems^1^. Among common major pollutants, synthetic dyes, produced at around 1 million tons per year with 20% entering water systems come from industries like textiles, leather, and cosmetics^2–5^. The most common type, azo dyes, resist degradation and cause DNA damage and cancer even at low levels, while their colors block sunlight and harm aquatic life^6,7^. One example, Acid Red 37 (AR-37), is widely used on wool, silk, and leather. The concentration of such reactive dyes in industrial wastewater can vary between 10 and 200 mg/L, depending on the specific textile and dyeing processes^8,9^.

Over years, various physical, chemical and biological methods are investigated for wastewater treatment, including adsorption, chemical precipitation, flocculation-coagulation, membrane filtration, ultrafiltration, electrodialysis, microbial degradation, photocatalysis, reverse osmosis and ion exchange^7,10,11^. Adsorption is still known as the most effective and widely used method due to its simplicity, cost-efficiency, and lack of hazardous by-products^12^. Effective adsorbents must be non-toxic, affordable, and abundant, with high adsorption rates and capacities. Adsorption capacity relies on surface area, pore size, and physical characteristics, where larger areas and functional groups improve active sites and efficiency^4,13^. Common candidates include activated carbon, synthetic or natural polymers, zeolites, biomass, and agricultural waste^14^. Among these, natural polysaccharide-based hydrogels feature 3D networks that absorb water while maintaining their structure^3,15^, serving in water treatment, food technology, biomedicine, and biosensor coatings^16^. Their effectiveness in trapping dyes is attributed to functional groups like –OH, –COOH, and –NH₂, which interact with various pollutants^17^. However, modification of adsorbents, including hybrid composite hydrogels, have achieved over 90% azo dye removal with capacities of 150–400 mg/g, for example chitosan-montmorillonite beads exceed 300 mg/g for methyl green adsorption^18^, while recent hybrid composite hydrogels attain 85–95% efficiency^11,19,20^. Chitosan (CS) and cellulose acetate (CA) are two widely used biodegradable polymers that have attracted increasing interest due to their environmental friendliness and safety for biological systems^17^. CS is modified natural polysaccharide derived from deacetylation of acetamide groups in chitin and is well-known for its low cost and antibacterial activity^21–23^. CA is a semi-synthetic derivative of cellulose, also offers low toxicity and is extensively applied in water and wastewater treatment due to its favorable physicochemical properties^24^. Recent studies indicate that CS offers superior adsorption capacity for dye pollutants and easier regeneration compared to many conventional adsorbents. For instance, a CS film achieved an adsorption capacity of 822.4 mg/g for reactive blue 19^3^, while a CS-spirulina composite membrane showed capacities of 120, 110, and 100 mg/g for methylene blue, lemon yellow, and reactive black 5, respectively^25^. Likewise, polyethylenimine-modified cellulose acetate fibrous membranes (CA-c-PEI) demonstrated a high Cr(VI) removal capacity of 285.7 mg/g, outperforming most traditional biomass-based materials^24^.

Despite the high adsorption capacity of Chitosan-based hydrogels, their instability in acidic conditions, softness and agglomeration tendency, hinder their performance^26,27^. Thus, chemical modifications, such as crosslinking could improve chitosan’s mechanical strength, reduce hydrophobicity, and increase stability at low pH^27,28^. The degree of crosslinking critically governs the elasticity, swelling, porosity, and mechanical strength of hydrogels. While essential for structural integrity, excessive crosslinking results in reduced flexibility and a less porous matrix, thereby decreasing sorption efficiency due to limited access to functional groups and chelating sites. Consequently, achieving an optimal and controlled crosslinking level is vital^29^.

Crosslinked chitosan materials have demonstrated improved adsorption performance and stability, with removal efficiencies reported above 85% under optimized conditions. Recent advancements in modifying chitosan have further enhanced their performance; for example, chitosan modified with polyacrylamide has demonstrated rapid adsorption, achieving 43% removal efficiency of methyl orange within only 10 min at pH 3.0^27^. Common crosslinkers include glutaraldehyde (GA), ethylene glycol diglyceryl ether, and epichlorohydrin^3,30^. Similarly, modification proceeded on the hydroxyl groups in cellulose acetate (CA) enhances its stability and increases its efficiency in water treatment^31^. The co-dissolution regeneration process is the most common method for preparing chitosan-based hydrogels^32,33^.

Recently, adsorption studies were merged with theoretical models such as DFT, providing valuable insights into the adsorbent/adsorbate interaction mechanisms, with certain simplifications. For instance, these models typically treat the hydrogel matrix as an idealized, homogeneous structure and may neglect the dynamic swelling behavior, cross-linking heterogeneity, and solvent-mediated interactions present in real systems. Additionally, the models are generally limited to static configurations, not fully capturing the time-dependent adsorption–desorption dynamics or the influence of pH and ionic strength fluctuations. Therefore, while theoretical results offer a molecular-level understanding of binding sites and interaction energies, that are usually interpreted as complementary to experimental findings rather than a complete representation of the complex adsorption process^34,35^. On the other hand, incorporating geometrical analysis of adsorbate/adsorbent system would provide deeper insight into the spatial arrangement, binding orientations, and structural adjustments taking place^36,37^. Such analysis is crucial for understanding how molecular geometry influences adsorption efficiency and interaction stability. While geometrical models inevitably simplify certain aspects of the real system, they offer a powerful means to bridge molecular-scale interactions with macroscopic adsorption behavior.

While previous studies have examined the effects of freeze-drying and air-drying on the porosity and adsorption performance of pure chitosan or other chitosan-based materials, to our knowledge, no systematic comparison of these two drying techniques has yet been reported for glutaraldehyde-crosslinked chitosan/cellulose acetate hybrid hydrogels^38,39^.

However, the novelty of the present work extends beyond a routine drying comparison. Specifically, we introduce three conceptual advances: (i) material design innovation: overcoming cellulose acetate’s historical limitation to 2D membranes by engineering a stable 3D crosslinked hydrogel with chitosan; (ii) structural stabilization theory: demonstrating for the first time that cellulose acetate acts as a ‘molecular scaffold’ preventing pore collapse during air-drying, thereby enabling cost-effective ambient-dried adsorbents; and (iii) predictive mechanistic modeling: integrating DFT and molecular dynamics to show that drying affects physical accessibility (pores) but not chemical affinity (active sites). Thus, the drying comparison serves to uncover fundamental material behavior, not an end in itself. The present work therefore investigates how freeze-drying versus air-drying influences the structural porosity and adsorption capacity of this unique crosslinked blend, while providing new design rules for biopolymer-based adsorbents.

In the present study, we address this gap by designing a sustainable chitosan/cellulose acetate hydrogel, crosslinked with a minimal amount of glutaraldehyde, and employing two distinct drying techniques as a strategic variable to engineer materials with divergent properties. Although greener crosslinkers (e.g., genipin, citric acid) are an active area of research, GA was selected for this foundational study due to its well-understood reaction kinetics and ability to produce a consistently robust network, allowing us to isolate and study the variable of drying method without interference from variable crosslinking efficiency. The resulting freeze-dried (CCA-HG_F_) and air-dried (CCA-HG_A_) hydrogels are not merely compared but are used to decisively demonstrate how processing dictate’s structure and function. A comprehensive suite of characterization techniques (FTIR, SEM, XRD, XPS, BET, swelling ratio, and point of zero charge) directly links the drying-induced structural differences to their performance in removing Acid Red 37 from aqueous solutions. Thus, batch adsorption experiments assess the influence of key parameters (pH, concentration, dose, time, temperature). Kinetic, isotherm, and thermodynamic analyses elucidate the adsorption mechanism and regeneration potential.Additionally, DFT and molecular dynamics simulations probe molecular interactions and electronic properties, providing atomistic validation. This multi-scale approach not only yields an efficient adsorbent but also delivers fundamental insights for rationally designing next-generation hydrogels for sustainable wastewater remediation.

## Materials and methods

### Materials and instrumentations

All materials and instruments used in this study for physicochemical and surface morphological analysis are described in the supplementary file. Materials were used referring to their MSDS sheets.

### Synthesis of chitosan/cellulose acetate hydrogels

Chitosan (2.00 g) was dissolved in 90.0 mL of acetic acid (1%) with continuous stirring at 45 °C for 2 h at 700 rpm. Cellulose acetate (0.50 g) was dissolved in 10.0 mL of glacial acetic acid while stirring at 50 °C for 1 h. Upon complete dissolution, the cellulose acetate solution was incorporated into the chitosan solution and mixed until homogeneous. Subsequently, glutaraldehyde (1.50 mL) (cross-linker) was added dropwise with continuous stirring until the hydrogel was formed (achieving the gelation point). Then the produced hydrogel was washed several times with distilled water to remove the excess acetic acid. To isolate air dried material (CCA-HG_A_), the hydrogel was left covered at room temperature (25 ± 2 °C) under normal atmospheric conditions for 7 days. For freeze drying (CCA-HG_F_), the hydrogel was first frozen at (–20 °C) for 24 h. to ensure complete solidification, then subjected to freeze-drying using a lyophilizer for 48 h.

The 4:1 ratio was selected as it provided the optimal balance between the high density of active adsorption sites (from chitosan) and the necessary structural reinforcement (from cellulose acetate), as higher concentrations of cellulose acetate were found to limit the swelling capacity and homogeneity of the resulting 3D network.

### Batch method for adsorption of AR-37

The adsorption performance of the isolated materials toward AR-37 was evaluated under varying pH, adsorbent dosage, initial dye concentration, contact time, temperature, foreign ions in addition to determination of point of zero charges of both materials. Detailed experimental procedures, including equations used for Removal percentage (R%) and adsorption capacity (q, mg/g) calculations, are provided in the Supplementary materials^21,40,41^.

### Regeneration and reusability

To evaluate the reusability of the hydrogels, dye-loaded hydrogels were collected after each batch adsorption experiment and left to dry. For the desorption process, a washing solution consisting of 60% ethanol and 1.0 M NaCl was prepared. Each hydrogel (CCA-HG_F_ and CCA-HG_A_) was treated separately by soaking in the solution for a suitable period under gentle agitation until the complete desorption of the Acid Red 37 dye was confirmed. The regenerated hydrogels were then thoroughly washed with distilled water multiple times until neutral pH and colorless filtrate were achieved, ensuring the complete removal of any remaining dye or desorbing agent. Subsequently, the regenerated hydrogels were reused in five adsorption–desorption cycles under the same predetermined optimum conditions to assess their regeneration ability^13^.

### Computational methodology

Innovative unit cell construction for the designed systems Na-Acid Red 37, hydrogel, and the Na-Acid Red 37/hydrogel adsorption complex was carried out using the Materials Studio Package^42^ for all DFT-based calculations. The computational setup employed the Perdew–Burke–Erzerhof (PBE) exchange–correlation functional within the generalized gradient approximation (GGA), enhanced with Grimme’s DFT-D3 semi-empirical dispersion correction to account for van der Waals interactions^43^. Projector–augmented wave (PAW) basis sets were used throughout the simulations^44,45^. A global orbital cutoff scheme was applied. The optimized geometries were expressed in Cartesian coordinates and further analyzed using GaussView software^46^ to extract electronic properties such as frontier molecular orbitals (FMOs)^47^. After geometry optimization, Forcite molecular dynamics simulations^48,49^ were performed at 2000 ps under an NVT ensemble using the universal force field to ensure reliable dynamic behavior. The Adsorption Locator module was used to identify the most favorable binding sites on the adsorption surface. using the Eq. 1, The adsorption energy of the system was calculated^50^.

1 $$\:{E}_{ads}={E}_{\raisebox{1ex}{$A$}\!\left/\:\!\raisebox{-1ex}{$H$}\right.}-\left({E}_{A}+{E}_{H}\right)$$

where E_A/H_​ is the total energy of the optimized Na-Acid Red 37/hydrogel complex, and E_A_ and E_H_ are the total energies of Na-Acid Red 37 and the hydrogel, respectively.

## Results and discussion

### Characterization of the synthesized hydrogels

The synthesis of CCA-HG was based on an optimized 4:1 chitosan-to-cellulose acetate mass ratio to achieve a synergistic balance between adsorption capacity and structural strength. This specific composition was selected to maximize the density of active amino (NH_2_) and hydroxyl (OH) groups provided by the chitosan backbone, while cellulose acetate (CA) serves as a reinforcing 3D scaffold to enhance mechanical stability in acidic media. Preliminary optimization trials, supported by literature on similar chitosan/cellulose derivative composites^51,52^, indicate that increasing the cellulosic content beyond this threshold leads to a ‘densification effect.’ This phenomenon is attributed to enhanced molecular entanglement and stronger hydrogen bonding between the polymer chains, which results in a more condensed and less flexible matrix. Consequently, this structural densification limits the internal porosity and restricts the effective diffusion of large dye molecules, such as AR-37, into the hydrogel’s interior. Therefore, the 4:1 ratio represents the optimal threshold for maintaining a high surface-to-volume ratio and effective active site accessibility while preventing structural collapse.

Characterization is based on comparison between main characteristics of the synthesized hydrogels before and after adsorption process, to achieve structure picture of the hydrogels and type of interaction with AR-37.

#### FT-IR analysis

**Fig. 1:**
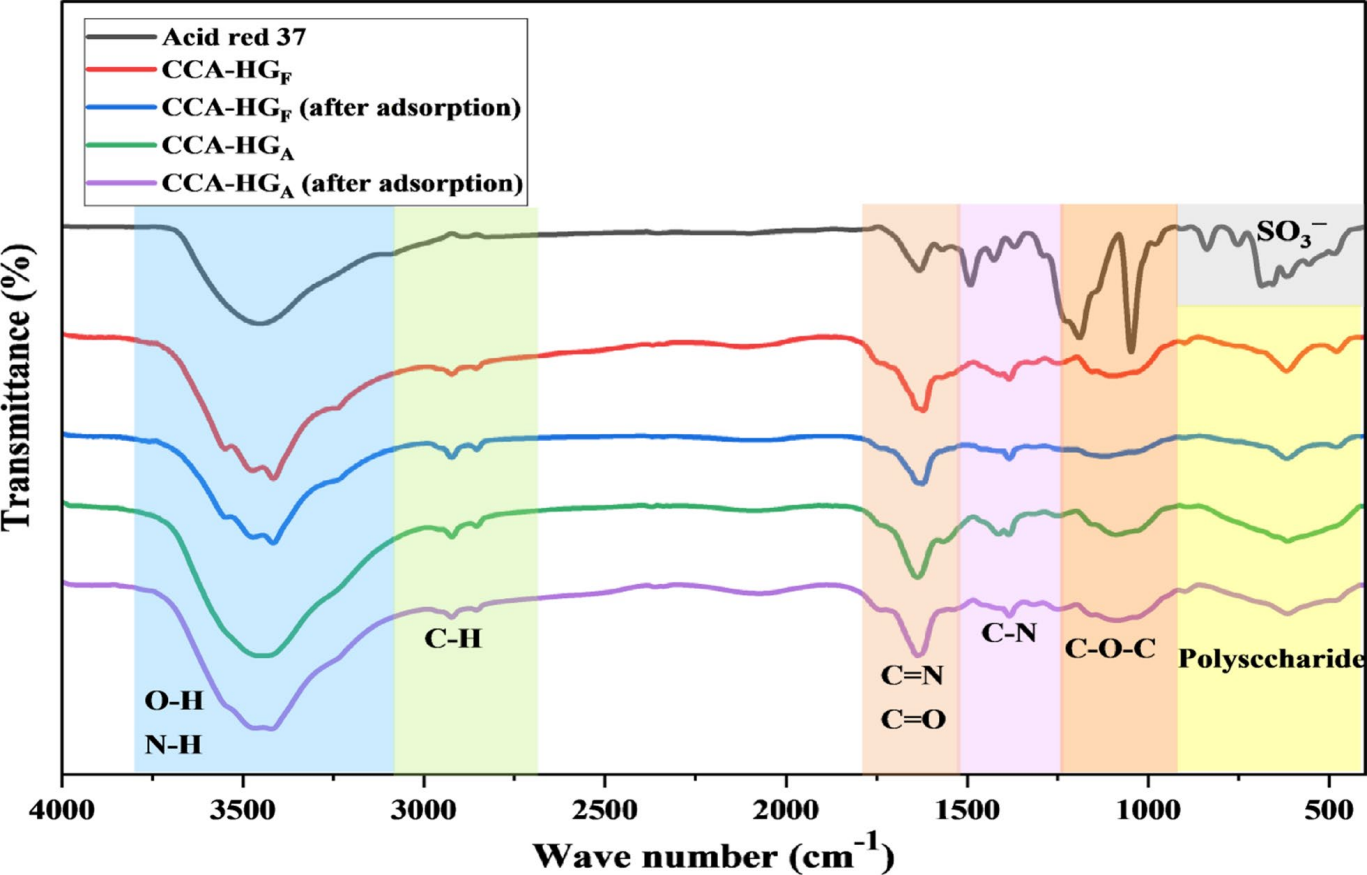
FTIR spectra of AR-37 and the synthesized hydrogels before and after adsorption.

FTIR spectra of the synthesized hydrogels (CCA-HG_F_ and CCA-HG_A_) are presented in Fig. 1. Comparing the spectra of CCA-HG_F_ and CCA-HG_A_ revealed some differences, mainly in the OH stretching region. The broad band at 3549 cm⁻¹ in CCA-HG_F_ corresponds to O–H stretching, reflecting hydrogen-bonded hydroxyl groups from both chitosan and cellulose acetate^25^. In CCA-HG_A_, the OH band appears even broader, which may be attributed to the presence of residual moisture entrapped within the polymer matrix (chitosan and cellulose acetate) due to air-drying occurred without vacuum. This interpretation considers both hydroxyl groups of chitosan and cellulose acetate and the influence of drying conditions on water retention. The N–H stretching of chitosan was observed at ~ 3415 cm⁻¹^53^, while the C–H aliphatic stretch appears at 2929 cm⁻¹, characteristic of cellulose acetate^54^. The band at 618 cm⁻¹, located in the fingerprint region, further supports the polysaccharide nature of the hydrogel material^55^.

A strong band at 1620 cm⁻¹ was observed in both samples, attributed primarily to the imine (C = N) bond, which indicates successful cross-linking between the amino groups of chitosan and glutaraldehyde. This band may also overlap with the carbonyl (C = O) stretching from cellulose acetate, but the shift in position suggests a modified chemical environment due to cross-linking^56^. The presence of a peak at 1384 cm⁻¹ corresponds to C–N stretching in chitosan^57^. Additionally, bands at 1240 cm⁻¹ and 1104 cm⁻¹ were assigned to C–O–C stretching, confirming the intact structure of cellulose acetate and the possible formation of acetal/hemiacetal linkages^58^.

After the adsorption of AR-37, the FTIR spectra exhibit distinct variations, particularly in the 1550–1400 cm^− 1^ and 1240 cm^− 1^ regions. By comparing the spectrum of the pure AR-37 dye with the loaded hydrogels, it is evident that the characteristic bands of the dye (such as the azo (N = N) and sulfonic (-SO_3_− groups)) now overlap with the functional groups of the hydrogel matrix. The noticeable shifts in these peaks serve as primary evidence of successful dye binding. These spectral overlaps and shifts strongly suggest synergistic interactions between the hydrogel’s active sites (amino, hydroxyl, and imine groups) and the dye’s functional moieties, confirming the effective entrapment and retention of AR-37 within the 3D hydrogel network.

#### Scanning electron microscope (SEM)

**Fig. 2:**
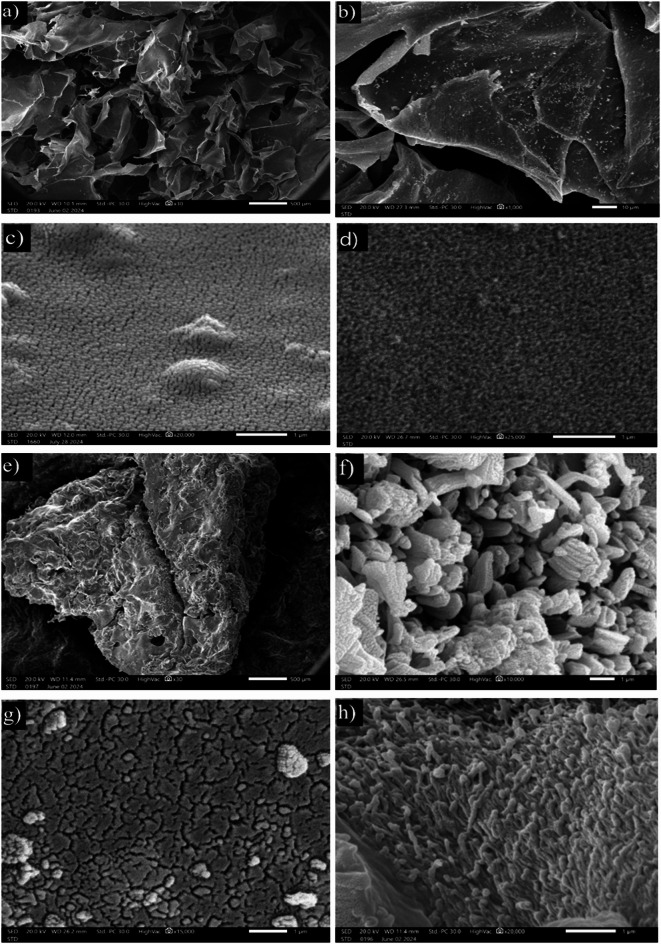
SEM images of CCA-HG_F_ (**a**,** b**,** c**,** d**) and CCA-HG_A_ (**e**,** f**,** g**,** h**).

The scanning electron microscopy images (SEM) of CCA-HG_F_ and CCA-HG_A_ are shown in Fig. 2 The primary difference between the two SEM images lies in the pore structure and overall morphology. The CCA-HG_F_ exhibits a highly porous and interconnected structure, while the CCA-HG_A_ has a more compact and less porous structure^33^. These differences can be attributed to the different drying methods used. Freeze-drying involves the rapid freezing of the hydrogel followed by the sublimation of the ice crystals, resulting in a minimal disruption of the hydrogel network and the preservation of the porous structure. In contrast, air-drying involves the gradual evaporation of water from the hydrogel, which may be the main reason for the significant shrinkage and collapse of the hydrogel network which increases its mechanical strength^38^.

#### XRD analysis

The XRD patterns for both CCA-HG_F_ and CCA-HG_A_ are shown in **Fig. S1**. Both CCA-HG_F_ and CCA-HG_A_ exhibit broad, amorphous peaks in their XRD patterns at approximately (2θ ≈ 28°), indicating a predominantly non-crystalline structure. There are no noticeable differences in peak intensity between the two samples, suggesting that neither the freeze-drying process nor the air-drying process significantly affected the crystallinity within the hydrogel structure^59^.

#### Surface area and porosity

**Fig. 3:**
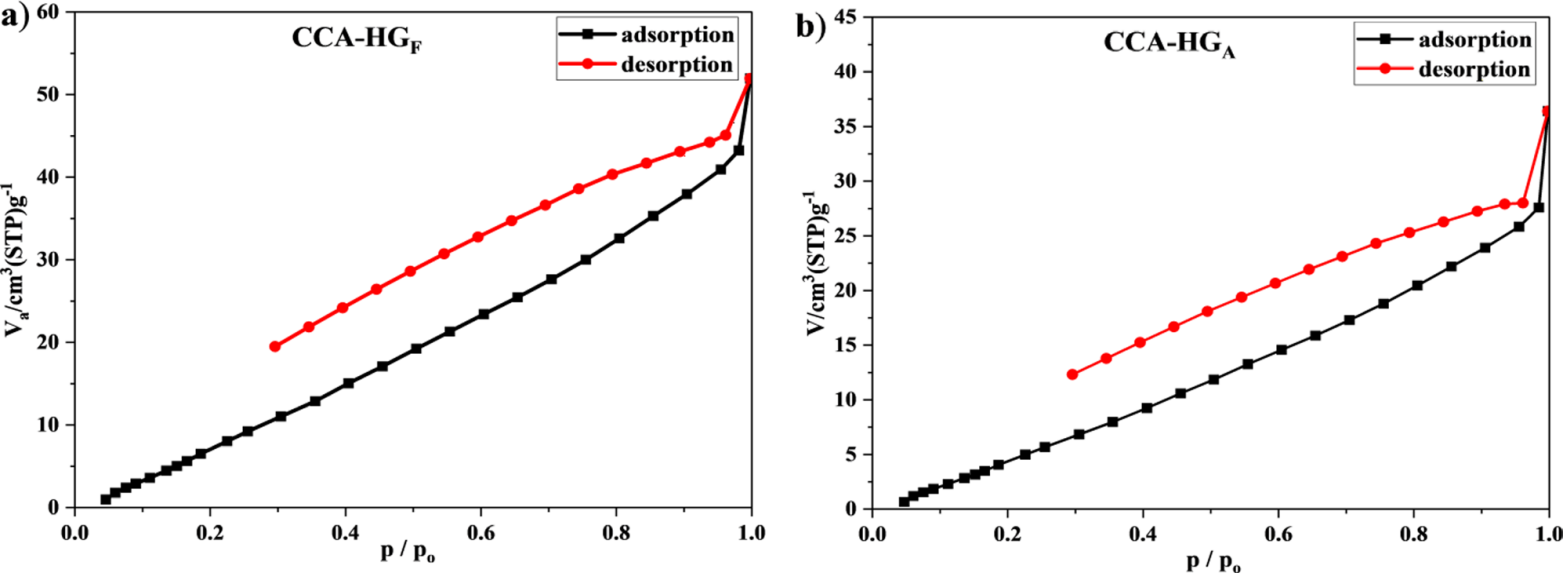
N_2_ adsorption-desorption isotherm of CCA-HG_F_ (**a**) and CCA-HG_A_ (**b**) hydrogels.

The data illustrated in Fig. 3; Table 1, are deduced from BET analysis. The N_2_ adsorption/desorption isotherms follow Type IV with an H2(b)hysteresis loop based on IUPAC classification, confirming the mesoporous nature of both CCA-HG_F_ and CCA-HG_A_ with irregular or complex pore structures^60^. CCA-HG_F_ possess significantly higher specific surface area and total pore volume compared to CCA-HG_A_. This suggests that the freeze-drying process results in a more porous structure. The higher specific surface area in the CCA-HG_F_ sample may be attributed to the rapid removal of water during the freezing process, which prevents the collapse of the pores. In contrast, CCA-HG_A_ shows reduced porosity and lower specific surface area due to the slower drying process, which leads to gradual pore collapse as water is removed over time, resulting in a denser structure compared to CCA-HG_F_^33^.

**Table 1:** BET surface area, pore volume, and average pore diameter of CCA-HG_F_ and CCA-HG_A_.

Sample	Bet surface area (m²/g)	Total pore volume (cm³/g)	Mean pore diameter (nm)
CCA-HG_F_	94.902	7.4123 × 10^− 2^	3.1241
CCA-HG_A_	52.757	4.8067 × 10^− 2^	3.6444

#### XPS analysis

**Fig. 4:**
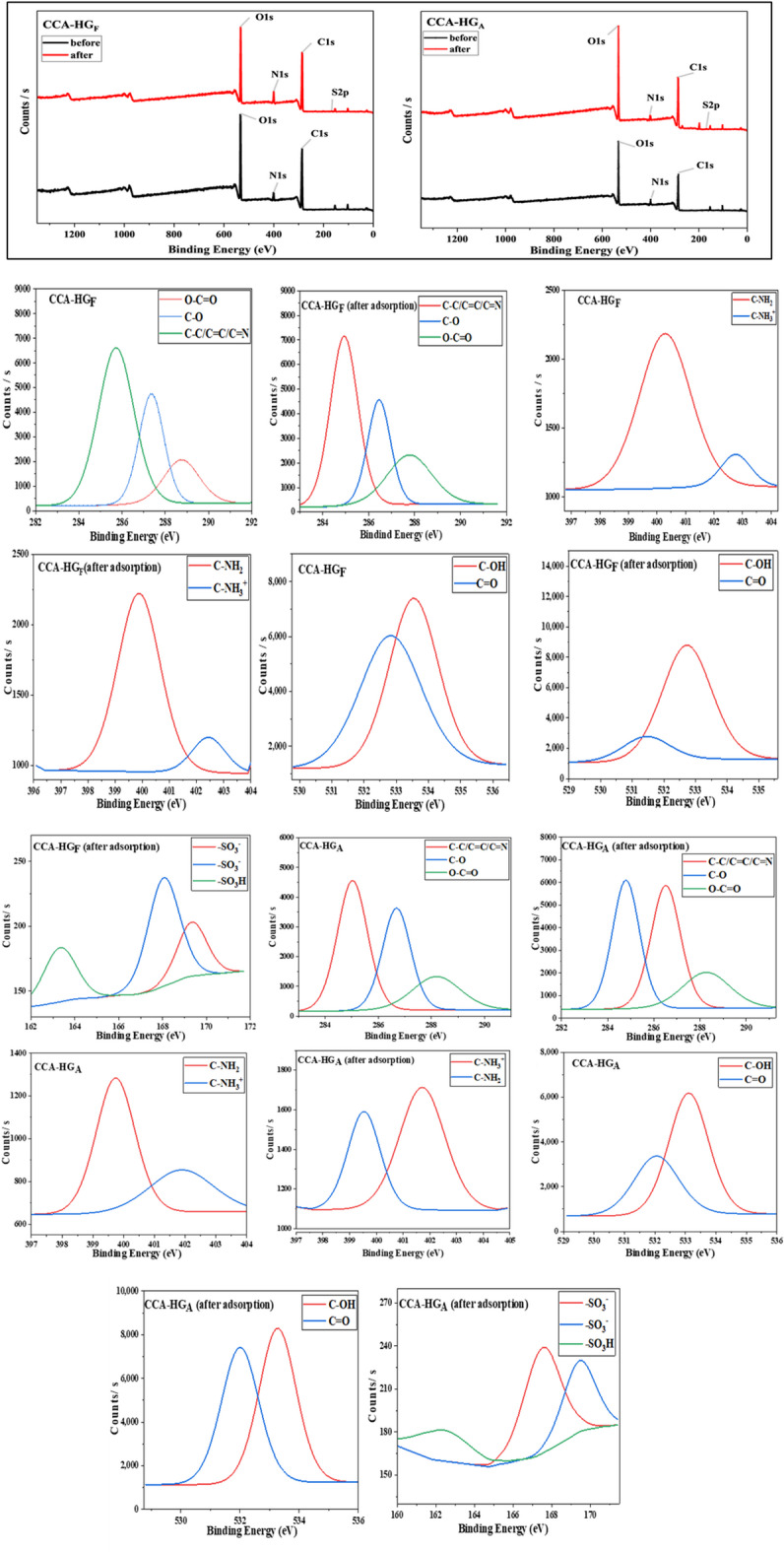
XPS spectra showing elemental composition of CCA-HG_F_ and CCA-HG_A_ before and after AR-37 dye adsorption.

Based on the XPS results, a description of main binding energies in both hydrogels, and a confirmation of successful Acid Red 37 adsorption was deduced by the comparison between the binding energy peaks before and after adsorption, corresponding to C1s, N1s, and O1s peaks, in addition to the dye- S2p new peak appearance after adsorption, Table 2; Fig. 4. The percentages of C1s, N1s, and O1s showed variation upon adsorption, in case CCA-HG_F_ changes from (66.93, 4.98, and 28.09) % into (68.84, 6.62, and 24.03) %, respectively, with S2p was found to be 0.24%, after adsorption. In case of CCA-HG_A_, three main peaks corresponding to carbon (C1s), oxygen (O1s), and nitrogen (N1s) were observed, with percentages of 65.96%, 28.88%, and 5.16%, respectively, before adsorption, and 65.55% for carbon, 29.73% for oxygen, and 4.61% for nitrogen, with the appearance of a sulfur (S2p) peak at 0.11%, after adsorption. The CCA-HG_F_ showed greater positive change indicating greater absorption. For CCA-HG_F_ before and after adsorption can be summarized as for the main C1s peaks (before/after) at (288.75/287.78)eV (287.36/286.46)eV, and (285.72/284.94) eV attributed to different environments around C as O-C = O, C-C, C = C, or C = N bonds from cellulose acetate, hydroxyl groups in chitosan and due to dye carbons that caused shifts and percent changes, highlighting a strong interaction between the dye and hydrogel functional groups during adsorption^39,61^. The main O1s peaks (before/after) at (533.82, 533.54/532.72, 531.45) eV which are attributed to hydroxyl (C-OH) and (C = O) groups originated from chitosan and cellulose acetate. The change in %O1s gives further evidence for the successful adsorption of AR-37 onto the hydrogel. The main N1s peaks at 400.28 and 402.76 eV corresponded to primary amines and protonated amines, respectively, showed shift after adsorption, to 399.88, and 402.44 eV, indicating alterations in the nitrogen bonding environment, reflecting interactions between the dye and the hydrogel^39^. Additionally, the appearance of characteristic S2p peaks at 168.87 and 168.15 eV, with ∆B.E.= 1.72 eV attributed to S as -SO_3_^−^ group and at 163.33 eV that may be attributed to its protonated form^62^. Similar performance appeared in case of air-dried hydrogel, CCA-HG_A_, with some shifts as shown Table 2. confirming the suggested structures of hydrogels and successful AR-37 dye adsorption.

**Table 2:** Interpretation of the XPS spectra of CCA-HG_F_ and CCA-HG_A_ before and after adsorption of AR-37.

Element	State	Binding energy (eV)		Binding energy (eV)	
		CCA-HG_F_		CCA-HG_A_	
		Before	After	Before	After
C1s	O-C = O	288.75	287.78	288.20	288.28
	C-O	287.36	286.46	286.69	286.52
	C-C/C = C/C = N	285.72	284.94	285.03	284.79
O1s	C-OH	533.54	532.72	532.88	532.73
	C = O	532.82	531.45	532.63	532.67
N1s	C-NH_2_	400.28	399.88	399.74	399.54
	C-NH_3_^+^	402.76	402.44	401.89	401.72
S2p	-SO_3_-	-	168.87	-	168.94
		-	168.15	-	167.83
		-	163.33	-	162.4

#### Swelling capacity

**Fig. 5:**
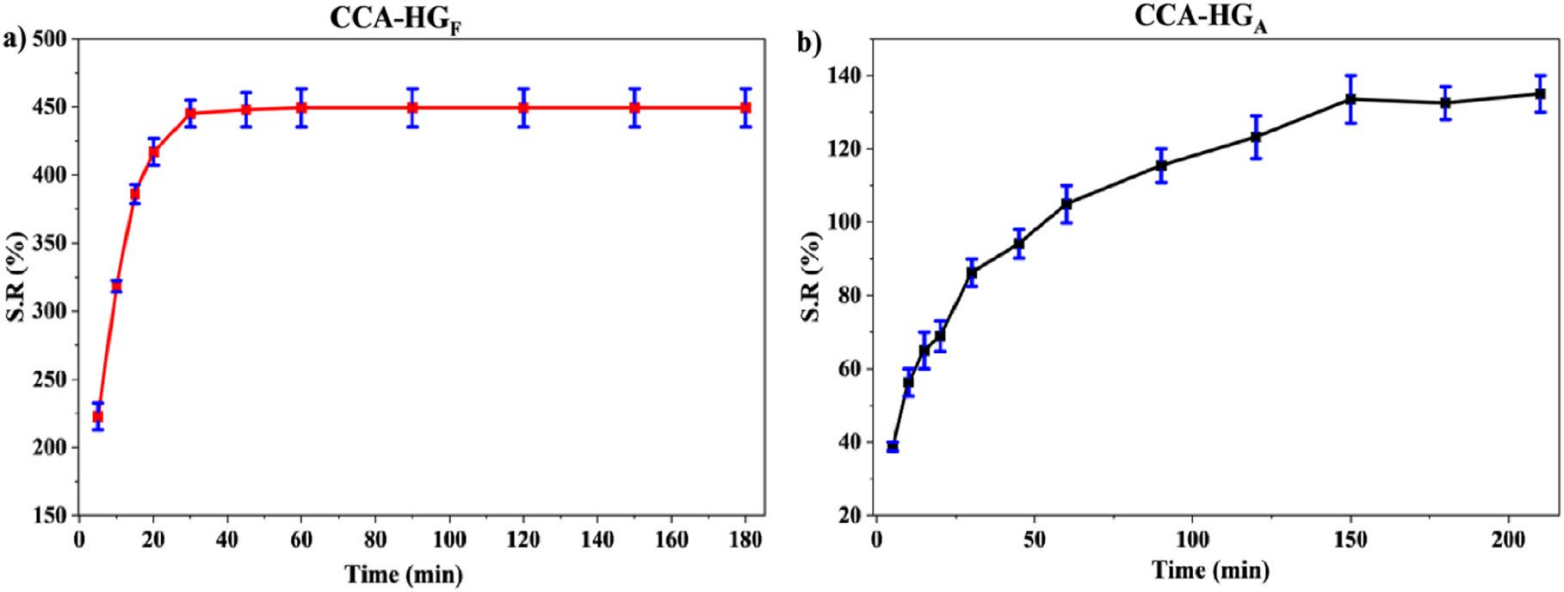
Swelling capacity profiles demonstrating the hydrogels absorption behavior.

The swelling behavior of the hydrogel was analyzed for both CCA-HG_F_ and CCA-HG_A_ in distilled water, providing insights into the porosity, structural stability, and suitability of the synthesized hydrogels, Fig. 5. A predetermined weight of each hydrogel sample was immersed in a sufficient volume of distilled water for a maximum duration of 150 min. Upon completion of the swelling period, the hydrogels were removed from the solution, and any excess water was gently removed using filter paper. The swelling ratio was then calculated using Eq. 2^26^.

2 $$Swelling\,ratio\,(\%) =\frac{Wt-Wd}{Wd}\times\:100$$

where *W*_*t*_ is the weight of the swollen hydrogel, and *W*_*d*_ is the initial dry weight of the hydrogel.

CCA-HG_F_ showed a significant increase in swelling, rising from 222% to 445% within a time extent of 5 to 30 min. This rapid swelling can be attributed to the highly porous structure created during the freeze-drying process, which facilitates faster water absorption and expansion of the hydrogel matrix. On the other hand, CCA-HG_A_ exhibited slower and more moderate swelling behavior. The swelling percentage increased from 39% to 134% over a longer period, from 5 to 150 min. The reduced swelling rate in air-dried samples is likely due to the denser and compact structure formed during the drying process (as indicated from SEM and surface area results) which limits water penetration and slows down the hydrogel’s expansion^29^. These results highlight the impact of the drying method on the hydrogel’s swelling capacity, with CCA-HG_F_ samples demonstrating faster and higher water uptake compared to CCA-HG_A_.

### Adsorption studies of acid red 37(AR-37) by freeze- and air-dried-hydrogels

Factors governing the adsorption efficiency; pH, Initial concentration, adsorbent dose, time, were studied, and optimum conditions were defined.

#### Point of zero charge

**Fig. 6:**
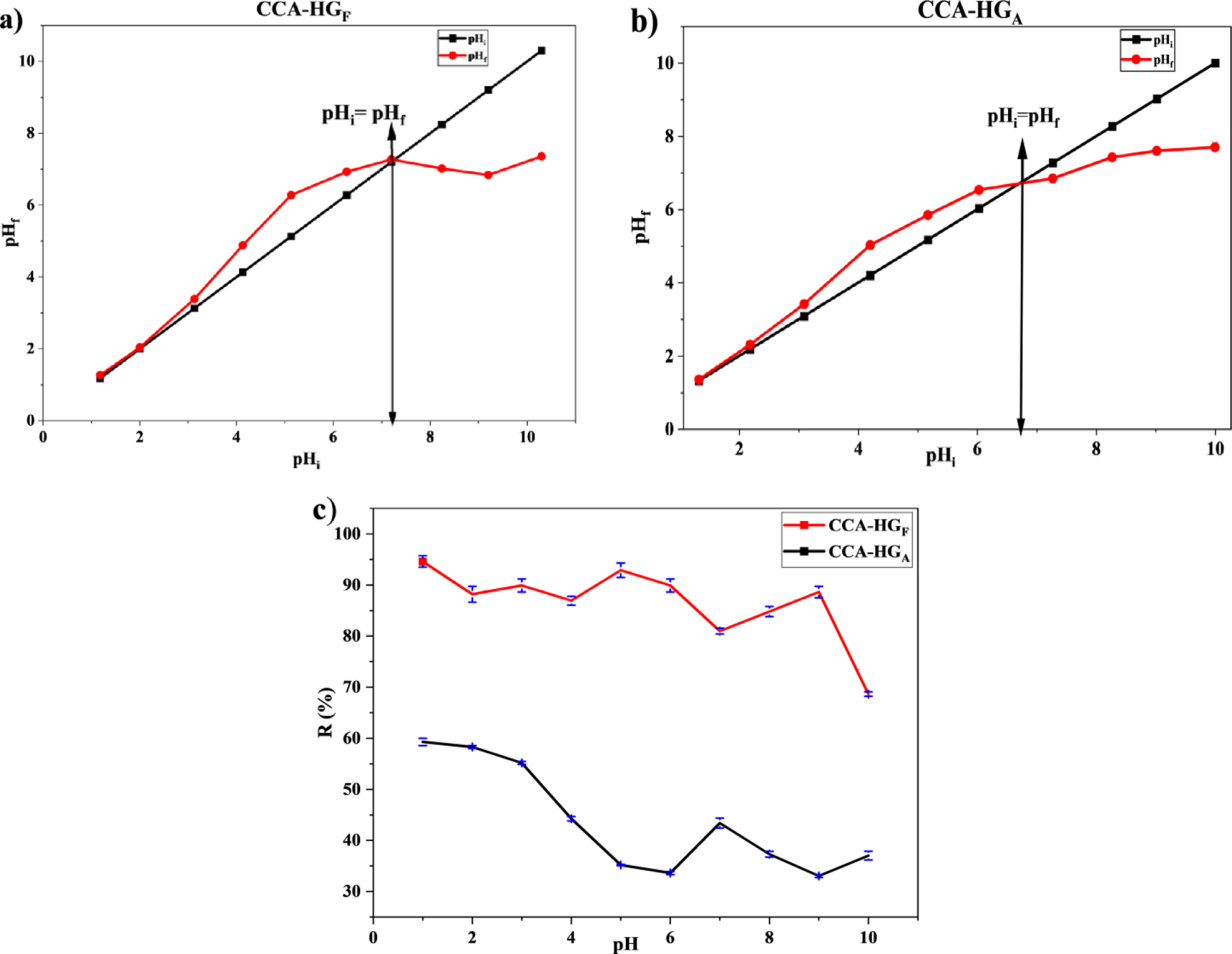
(**a**,** b**) Point of zero charge (pHpzc) of CCA-HG_F_ and CCA-HG_A_, and (**c**) Effect of pH on AR-37 dye adsorption onto CCA-HG_F_ and CCA-HG_A_.

The pH_PZC_ was defined using the pH drift method. As shown in Fig. 6a, b, The intersection of the two curves indicates a pH_PZC_ of 7.35 and 6.75 for CCA-HG_F_ and CCA-HG_A_, respectively, meaning the surface has a net positive charge at lower pH values and a net negative charge at higher pH values. When the solution pH was below this point, the removal efficiency of AR-37 dye was relatively high, likely due to the increased presence of positive charges on the carbon surface^63^.

The observed difference of 0.6 units between the two hydrogels can be primarily attributed to the influence of the drying methods on the surface functional groups’ accessibility and structural integrity. Freeze-drying preserves the porous structure by minimizing capillary forces, thus maintaining the availability of basic functional groups such as –OH and –NH₂ for protonation, which contributes to a slightly higher pH_PZC_ of CCA-HG_F_. In contrast, air-drying tends to induce pore shrinkage or collapse due to gradual water evaporation, potentially rearranging or screening functional groups and reducing their protonation capacity, leading to a slightly lower pH_PZC_ for CCA-HG_A_^38^. Nevertheless, both values remain within a comparable range, supporting efficient dye adsorption under acidic conditions.

#### Effect of pH

The pH of the solution significantly influences the adsorption capacity of adsorbates such as, Acid Red 37 (AR-37) dye by affecting both the surface charge of the adsorbent or the ionization of the dye. As illustrated in Fig. 6, the impact of pH on the adsorption of AR-37 by CCA-HG_F_ and CCA-HG_A_ was examined across a pH range of 1 to 10. Within the experimental conditions, described in section (S1.3.), the highest removal efficiency of 94.61% and 59.30% for CCA-HG_F_ and CCA-HG_A_, respectively, at pH 1, (below PZC), where the surface positive charge enhances electrostatic interactions with the negatively charged dye molecules, resulting in more efficient adsorption^14^. This behavior is attributed to the ionization state of the amino groups in the chitosan chains, which have a pK_a_ value of approximately 6.5. At pH levels below this pK_a_, these amino groups are highly protonated (-NH_3_^+^), creating a dense positive surface charge. This enhances electrostatic interactions with the negatively charged AR-37 dye molecules, resulting in more efficient adsorption^64,65^. Although such extremely acidic conditions may not be common in real textile effluents, the experiments at pH 1 were performed to explore the maximum adsorption capacity and to understand the adsorption mechanism under highly protonated environments. Significantly, CCA-HG_F_ maintained a relatively stable and high removal efficiency (above ~ 85%) from pH 1 to 7, while CCA-HG_A_ showed a gradual decrease over the same range but still retained practical performance, indicating that the hydrogels can still perform effectively under more typical wastewater conditions.

Notably, CCA-HG_F_ maintains relatively high adsorption efficiency even at higher pH values, where both the hydrogel surface and the dye molecules are negatively charged, which typically induces electrostatic repulsion. This sustained performance is attributed to its open porous structure (resulting from freeze-drying), which facilitates dye diffusion and allows non-electrostatic interactions, such as hydrogen bonding, π–π stacking, and van der Waals forces to play a significant role in the adsorptoin^66^.

#### Effect of adsorbent dose

**Fig. 7:**
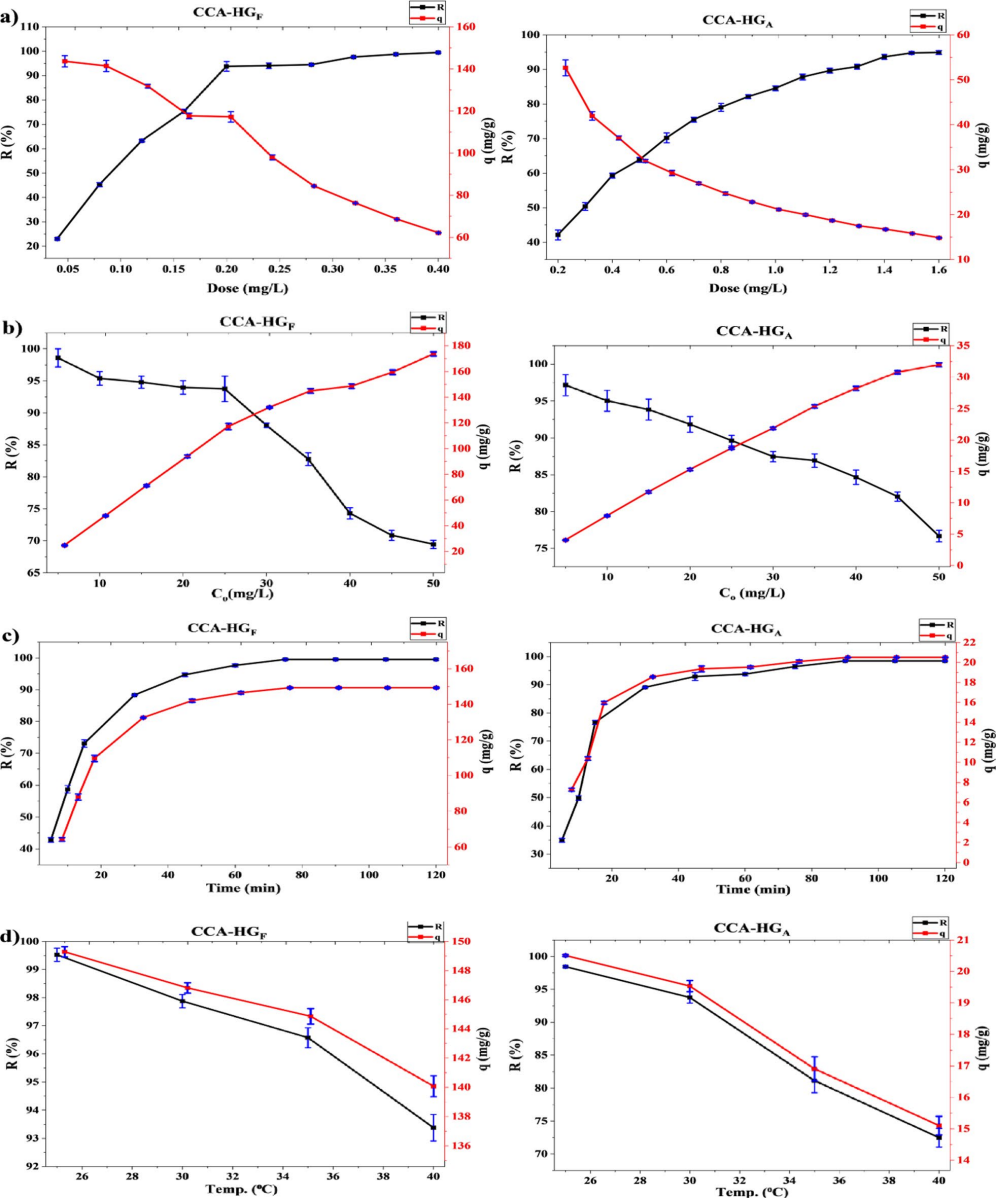
Effect of (**a**) adsorbent dose (0.05–1.6 g/L), (**b**) initial AR-37 dye concentration (5–50 mg/L), (**c**) contact time (5–120 min), and (**d**) temperature (25–40 °C) on the adsorption capacity (q) and the removal percentage (R) of AR-37 dye using CCA-HG_F_ and CCA-HG_A_).

The impact of synthesized hydrogels’ dose on AR-37 dye adsorption was examined with dosages ranging from 0.04 to 0.4 g/L for CCA-HG_F_ and from 0.08 to 1.6 g/L for CCA-HG_A_. The results demonstrated an increase in removal efficiency with the rising adsorbent dose, as shown in Fig. 7a, for CCA-HG_F_, the removal efficiency increased from 23% to 99%, while CCA-HG_A_ improved from 42% to 95%. This enhancement is attributed to the greater availability of active sites, allowing for more binding with AR-37 dye molecules. At a constant dye concentration (25.0 mg/L), the adsorption capacity (q) decreased as the dye spread over a larger surface area, while the removal efficiency (R%) initially increased and then slowed down as the system approached equilibrium^67^. Therefore, the optimum doses were selected as 0.20 g/L for CCA-HG_F_ and 1.20 g/L for CCA-HG_A_.

#### Effect of initial concentration of AR-37

To investigate the effect of initial dye concentration on the removal efficiency of CCA-HG_F_ and CCA-HG_A_, concentrations ranging from 5 to 50 mg/L were selected with the predetermined optimum pH and dose at room temperature, for 30 min. As shown in Fig. 7b the removal efficiency (R%) and adsorption capacity (q) were plotted against the initial dye concentration (C_o_). The results show that as dye concentration increases, removal efficiency decreases (from 100% to 70% for CCA-HG_F_ and from 97% to 77% for CCA-HG_A_) due to the limited active sites on the adsorbent. At lower concentrations, the active sites effectively bind dye molecules, ensuring high efficiency. However, as concentration rises, saturation occurs, reducing efficiency. In contrast, adsorption capacity increases with higher concentrations because the stronger driving force overcomes mass transfer resistance^21,68^. To achieve a balance between removal efficiency and adsorption capacity, AR-37 dye concentrations of 30 and 25 mg/L were selected as optimum initial concentrations for CCA-HG_F_ and CCA-HG_A_, respectively.

#### Effect of contact time

In examining the effect of contact time on the removal efficiency of AR-37, time intervals ranging from 5 to 120 min were investigated, as illustrated in Fig. 7c at the predetermined optimum pH, dose and initial concentration at room temperature. The results indicated a significant increase in removal efficiency over time; for CCA-HG_F_, it rose sharply from 43% to 99.53% between 5 and 75 min, while for CCA-HG_A_, it increased from 35% to 98.44% between 5 and 90 min. This initial rapid adsorption may be attributed to the abundance of available active sites and the high dye concentration. As the process progressed, the active sites became saturated with the dye (at 70–120 min for CCA-HG_F_ and 90–120 min for CCA-HG_A_), leading to a decrease in available active sites and ultimately reaching equilibrium^69^.

#### Effect of temperature

To investigate the effect of temperature on the adsorption of AR-37 dye, a range from 25 °C to 40 °C was studied at optimum conditions. The results shown in Fig. 7d, indicate a slight decrease in removal efficiency as the temperature increased, for CCA-HG_F_ (from 99.53% to 93.38%), while the decrease was more pronounced for CCA-HG_A_ (from 98.44% to 72.48%), likely due to reduced interactions between dye molecules and the adsorbent caused by higher kinetic energy. The highest adsorption performance was achieved at room temperature (25 °C). Therefore, this temperature was selected as the optimum temperature for further studies^70^.

#### Effect of foreign ion

**Fig. 8:**
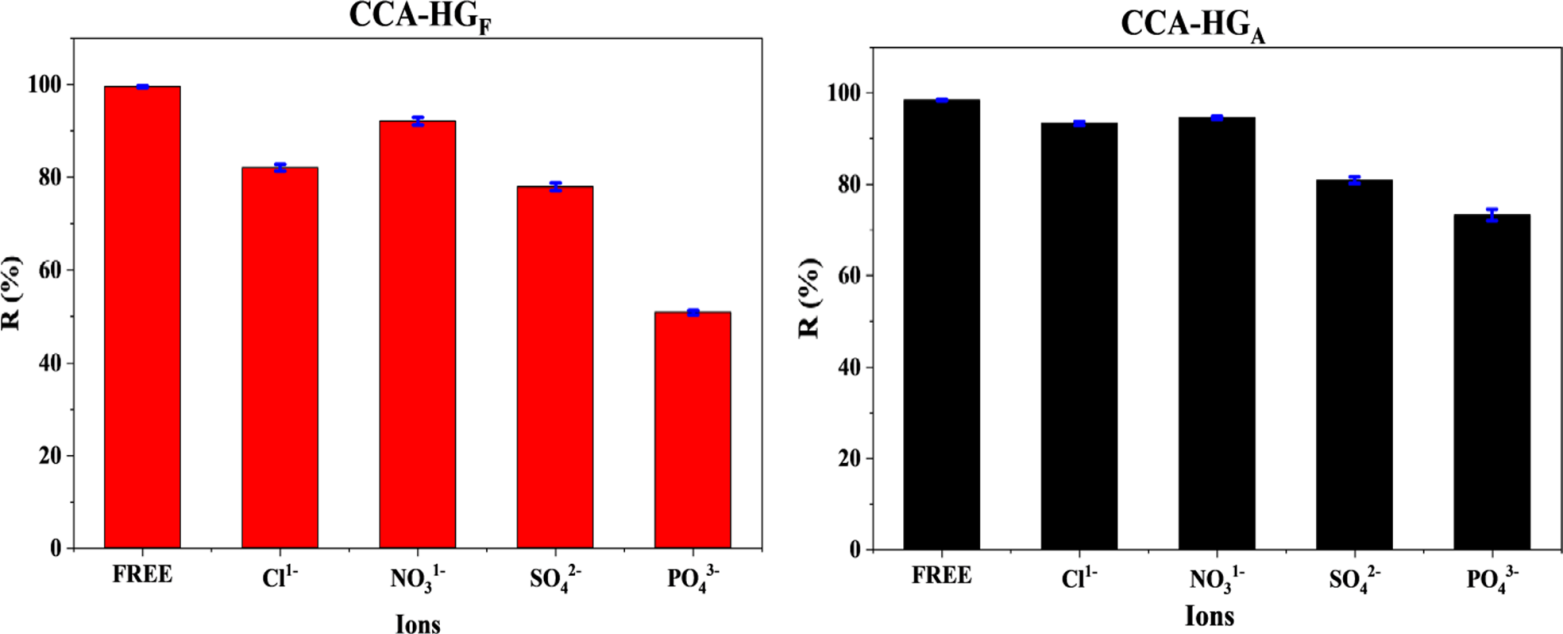
Effect of foreign ions on the removal percentage (R) of AR-37 adsorption by CCA-HG_F_ and CCA-HG_A_.

The presence of various anions in real water environments can significantly influence the adsorption behavior of AR-37 dye. As shown in Fig. 8, most of the tested anions (Cl⁻, NO₃⁻, SO₄²⁻) caused only a slight to moderate decrease in the dye removal efficiency, indicating limited interference with the adsorption process. However, the phosphate ion (PO₄³⁻) exhibited a more pronounced negative impact. This can be attributed to the high negative charge density of the phosphate ion compared to the other tested anions, which enhances its electrostatic attraction to the positively charged sites on the adsorbent surface. Moreover, due to its smaller size relative to the AR-37 dye, phosphate ions can access and occupy active adsorption sites, leading to stronger competitive sorption^41^. As a result, the hydrogel showed a higher affinity toward phosphate ions than AR-37 dye causing a significant drop in removal efficiency.

### Kinetics modeling

**Fig. 9:**
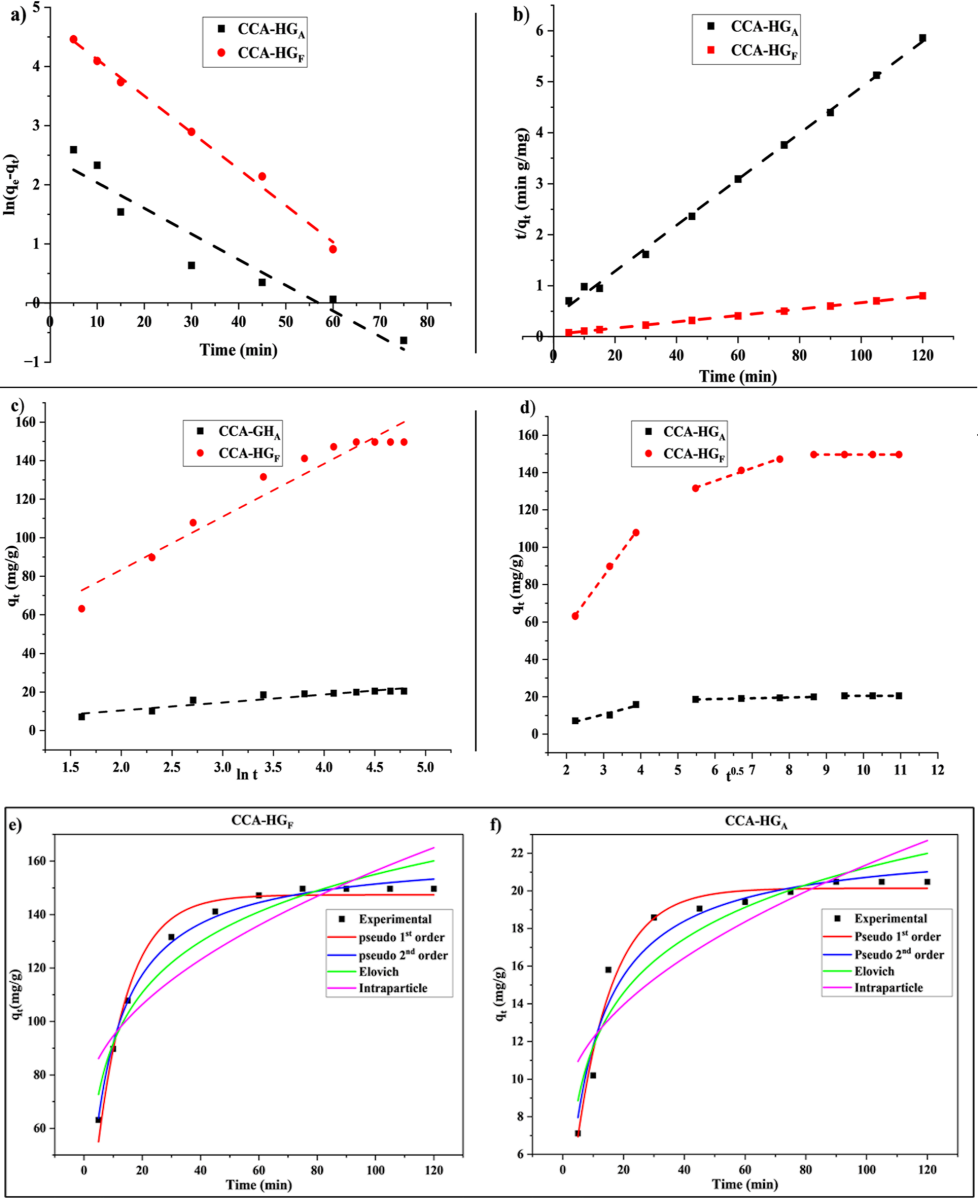
Linear fitting plots of the (**a**) pseudo-first order, (**b**) pseudo-second order, (**c**) Elovich model, (**d**) Intra-particle diffusion model and nonlinear fitting (**e**,** f**) for the adsorption of AR-37 onto CCA-HG_F_ and CCA-HG_A_.

The adsorption kinetics of AR-37 dye onto CCA-HG_F_ and CCA-HG_A_ were evaluated using linear and nonlinear fitting of four kinetic models: pseudo-first-order, pseudo-second-order, Elovich, and intra-particle diffusion. These models are illustrated in Fig. 9, and the corresponding calculated results are presented in Table 3. equations used for examined kinetic models are collected in Table S1. The pseudo-first-order model, Fig. 9a, assuming that adsorption is primarily a physical process that depends on the initial dye concentration. The correlation coefficients of this model (R²) values are 0.925 and 0.994 for CCA-HG_F_ and CCA-HG_A_, respectively.

The pseudo-second-order model, Fig. 9b, assumes that the adsorption process is mainly controlled by chemisorption. Both CCA-HG_F_ and CCA-HG_A_ showed an excellent fit to the **pseudo-second-order kinetic model**, with high R^2^ values of 1. This strong correlation is consistent across all comparable studies shown in **Table S4** and suggests that the rate-limiting step for the adsorption of AR-37 is primarily governed by a chemisorption mechanism. The kinetic rate constant (K_2_) for CCA-HG_A_ (0.005 g/mg.min) is notably higher than that of CCA-HG_F_ (0.001 g/mg.min) and other similar adsorbents, indicating a faster initial adsorption rate for CCA-HG_A_, despite its lower equilibrium capacity. The calculated q_e_ values (161.29 mg/g and 22.17 mg/g) closely aligned with the experimental values (149.30 mg/g and 20.50 mg/g), further supporting the model’s applicability^71,72^. The Elovich model, Fig. 9c, often applied to systems with heterogeneous active sites and varying adsorption energies, showed relatively low fitting with produced R² values of 0.895 and 0.943 for CCA-HG_F_ and CCA-HG_A_, respectively, suggesting that it may not fully capture the adsorption mechanism.

The intra-particle diffusion model, Fig. 9d, considering adsorption as possibly controlled by particle diffusion, characterizes the process in three stages: a rapid initial adsorption onto the external surface of the adsorbents, a slower diffusion into the pores, and the attainment of equilibrium. Since the graph did not intersect the origin, it suggests that intra-particle diffusion is not the sole rate-limiting step in the overall adsorption process^71,73^.

In summary, the pseudo-second-order model provided the highest R² values and produced q_e_ values closest to the experimental data, identifying it as the most suitable model for describing the adsorption kinetics of AR-37 dye onto CCA-HG_F_ and CCA-HG_A_. This suggests that the adsorption mechanism is predominantly driven by chemical interactions between the dye and the hydrogel materials.

To further confirm the accuracy of the results, nonlinear fitting was applied. As shown in Fig. 9(e, f), The pseudo-first-order model also provided an excellent description, with calculated q_e_ values (147.4 and 20.1 mg/g) almost identical to the experimental results, and R² = 0.98 for both adsorbents. This indicates that while chemisorption may play a significant role in the adsorption rate, suggested by the linear approach, the nonlinear analysis demonstrates that physical adsorption cannot be excluded. Overall, applying both linear and nonlinear fitting strengthens the reliability of the kinetic interpretation and highlights the nature of the adsorption process.

**Table 3:** Kinetic parameters of AR-37 adsorption onto CCA-HG_F_ and CCA-HG_A_.

Model	Parameter	Linear fitting		Nonlinear fitting	
		CCA-HG_F_	CCA-HG_A_	CCA-HG_F_	CCA-HG_A_
	q_e, exp_ (mg/g)	149.30	20.50	149.30	20.50
Pseudo-first order	R^2^	0.994	0.925	0.98	0.98
	q_e, cal_ (mg/g)	114.17	11.83	147.37	20.14
	K_1_ (min^− 1^)	0.062	0.043	0.093	0.084
Pseudo-second order	R^2^	1	1	0.995	0.957
	q_e, cal_ (mg/g)	161.290	22.173	163.20	22.632
	K_2_ (g/mg min)	0.001	0.005	0.00079	0.0048
Elovich	R^2^	0.943	0.895	0.943	0.895
	Β (g/mg)	0.036	0.242	0.036	0.242
	Α	77.191	7.034	28.39	2.59
Intra-particle diffusion	R^2^	0.818	0.761	0.82	0.76
	K_id_ (mg/g min^1/2^)	9.0477	1.346	9.05	1.34
	C (mg/g)	65.885	7.931	65.88	7.93

### Adsorption isotherm study

**Fig. 10:**
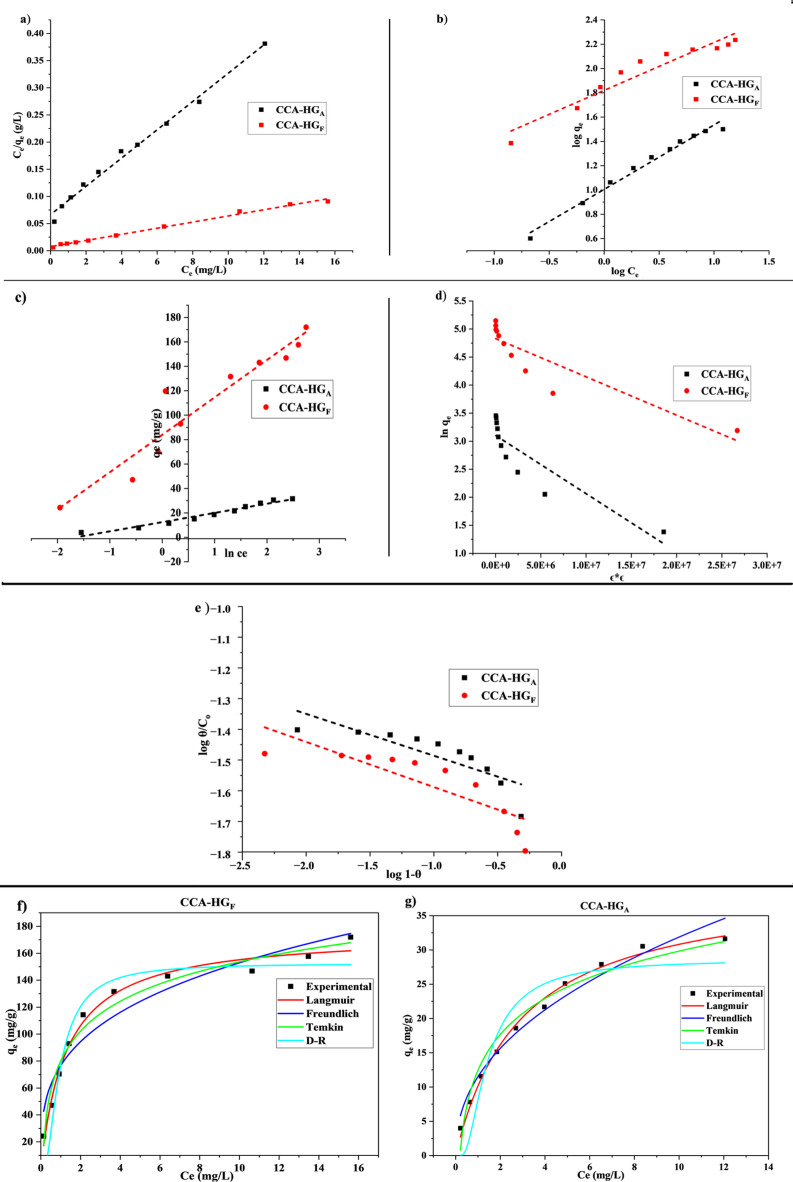
Adsorption isotherm plots and their corresponding fittings of AR-37 over CCA-HG_F_ and CCA-HG_A_ based on the linear forms of Langmuir (**a**), Freundlich (**b**), Temkin (**c**), Dubinin-Radushkevich (**d**), Flory-Huggins (**e**) and nonlinear fitting (**f**,** g**).

To optimize the adsorption process for eliminating AR-37 from aqueous solutions, various linear and nonlinear isotherm models were applied, including Langmuir, Freundlich, Temkin, Dubinin–Radushkevich (D–R), and Flory-Huggins, as illustrated in Fig. 10 and summarized in Table 4. The corresponding equations are presented in Table S2.

Among the tested models, the Langmuir isotherm (Fig. 10a) exhibited the best fit, with high R² values (0.99 for both CCA-HG_F_ and CCA-HG_A_),this finding is consistent across all comparable studies on chitosan-based adsorbents, as shown in Table S4, suggesting a monolayer adsorption mechanism on a homogenous surface. Notably, the maximum adsorption capacity (q_max_) of CCA-HG_F_ (175.44 mg/g) was significantly higher than that of CCA-HG_A_ (38.46 mg/g), while the binding affinity constant (K_l_) also favored CCA-HG_F_ (0.75 vs. 0.39), confirming its stronger interaction with the adsorbate^74^.

The Freundlich model (Fig. 10b) also provided a good fit, especially for CCA-HG_A_ (R² = 0.98), and a slightly lower correlation for CCA-HG_F_ (R² = 0.92). The adsorption strength (n) values of 2.54 (CCA-HG_F_) and 1.89 (CCA-HG_A_) suggest favorable adsorption on heterogeneous surfaces for both hydrogels^75^.

The Temkin isotherm (Fig. 10c) yielded R² values of 0.92 for CCA-HG_F_ and 0.96 for CCA-HG_A_, suggesting the influence of adsorbent–adsorbate interactions. The heat of adsorption (B_t_) values was 30.62 J/mol for CCA-HG_F_ and 7.54 J/mol for CCA-HG_A_, indicating a linear decrease in adsorption energy as surface coverage increases^76^.

The Dubinin–Radushkevich (D–R) model (Fig. 10d) showed a moderate correlation (R² ≈ 0.80 for both hydrogels), supporting a pore-filling mechanism. The calculated adsorption energy (E) values were 2.67 kJ/mol for CCA-HG_F_ and 2.24 kJ/mol for CCA-HG_A_, confirming the occurrence of physisorption^74,76^.

In contrast, the Flory–Huggins model (Fig. 10e) showed relatively low R² values (0.72 for CCA-HG_F_ and 0.69 for CCA-HG_A_), indicating a weaker fit. However, the negative Gibbs free energy change (ΔG) values of − 3.82 kJ/mol (CCA-HG_F_) and − 4.08 kJ/mol (CCA-HG_A_) confirm that the adsorption process is spontaneous^75^.

In conclusion, the combined insights from the Langmuir and Freundlich models suggest that adsorption onto both CCA-HG_F_ and CCA-HG_A_ involves a mixture of monolayer and multilayer mechanisms. Additionally, the Temkin and D–R isotherms confirm that the process is dominated by physisorption. Overall, CCA-HG_F_ demonstrated significantly higher adsorption capacity and stronger interaction with AR-37 compared to CCA-HG_A_ across all models.

In addition, the nonlinear fitting results, Fig. 10(f, g) were in strong agreement with the linear models. Both approaches confirmed the Langmuir model as the best fit (R² = 0.99) and nearly identical qmax values (≈ 175 mg/g and 39.81 mg/g for both adsorbents. Similarly, Freundlich, Temkin, and D–R models maintained consistent interpretations, supporting the coexistence of monolayer and multilayer adsorption as well as the dominance of physisorption. This agreement highlights the reliability of the obtained isotherm parameters and confirms the validity of the adsorption mechanism.

**Table 4:** Adsorption isotherms linear and nonlinear fitting data, calculation, and their parameter.

Model	Parameter	Linear fitting		Nonlinear fitting	Nonlinear fitting
		CCA-HG_F_	CCA-HG_A_		
Langmuir	R^2^	0.99	0.99	**CCA-HG**_**F**_	**CCA-HG**_**F**_
	K_L_ (L/mg)	0.75	0.39	0.99	0.99
	q_max, cal_ (mg/g)	175.44	38.46	0.77	0.77
Freundlich	R^2^	0.92	0.98	175.36	175.36
	K_F_ (mg/g) (L/mg)^1/n^	66.12	10.13	0.92	0.92
	n	2.54	1.89	76.68	76.68
Temkin	R^2^	0.92	0.96	3.34	3.34
	B_T_ (J/mol)	30.62	7.54	0.97	0.97
	b (J/mol)	80.90	328.4	31.97	31.97
	K_T_ (L/mg)	15.52	5.21	77.5	77.5
Dubinin-Radushkevich	R^2^	0.80	0.79	12.2	12.2
	E (kJ/mol)	2.67	2.24	0.93	0.93
	q_max, cal_ (mg/g)	125.39	22.28	1.463	1.463
Flory-Huggins	R^2^	0.72	0.69		
	∆G (kJ/mol)	−3.82	−4.08		

### Thermodynamic study

**Fig. 11:**
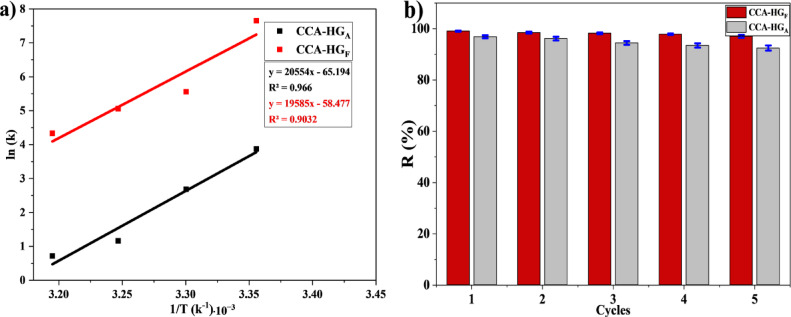
(**a**) Representation of thermodynamic curve for the removal of AR-37, (**b**) Reusability of CCA-HG_F_ and CCA-HG_A_ for the adsorption of AR-37 over five successive reusability cycles. The experiments were conducted under predetermined optimum conditions.

Thermodynamic parameters, including Gibbs free energy (ΔG°), enthalpy (ΔH°), and entropy change (ΔS°) were evaluated using the thermodynamics’ equations (collected in Table S3), to assess the feasibility of the adsorption process, Fig. 11a. These parameters were estimated at four different temperatures: 25 °C, 30 °C, 35 °C, and 40 °C, and the results are summarized in Table 5. The ΔG° values for CCA-HG_F_ and CCA-HG_A_ were both negative, indicating that the adsorption of AR-37 onto the synthesized hydrogels occurs spontaneously. The ΔH° values for both hydrogels were also negative. It suggests that the process is highly **exothermic** and is favored at lower temperatures.These values are significantly higher than those observed in other chitosan-based hydrogels (Table S4), indicating a much stronger bond formation between AR-37 and the hydrogels developed in this work. This can be attributed to the strong electrostatic attraction between the anionic dye and the positively charged hydrogel surface under acidic conditions, which leads to energy release during the adsorption. This result supports the proposed adsorption mechanism^77^. Additionally, the ΔS° values were negative for both CCA-HG_F_ and CCA-HG_A_, show that AR-37 dye molecules bind to certain active sites on the hydrogel adsorbents in an organized way, which makes things less random. The exothermic character of the adsorption (negative ΔH°) makes up for this ordering, which keeps the process spontaneous as a whole (negative ΔG°)^78,79^.

**Table 5:** Thermodynamic parameters for AR-37 adsorption onto CCA-HG_F_ and CCA-HG_A_.

Temperature (K)	ΔG° (kJ/mol)		ΔH° (kJ/mol)		ΔS° (kJ/mol.K)	
	CCA-HG_F_	CCA-HG_A_	CCA-HG_F_	CCA-HG_A_	CCA-HG_F_	CCA-HG_A_
298	−17.95	−9.36	−162.83	−170.89	−0.49	−0.54
303	−15.52	−6.65				
308	−13.09	−3.94				
313	−10.66	−1.23				

### Regeneration and reusability

The ability to regenerate adsorbents is essential for their economic and environmental feasibility. In this study, a 60% ethanol and 1.0 M NaCl solution was used to remove the adsorbed Acid Red 37 dye onto both CCA-HG_F_ and CCA-HG_A_, followed by thorough rinsing with distilled water. The regenerated hydrogels were reused over five adsorption-desorption cycles under optimum conditions, with results shown in Fig. 11b. Both CCA-HG_F_ and CCA-HG_A_ maintained consistently high removal efficiencies throughout the five regeneration cycles, with only minor variations observed. The CCA-HG_F_ hydrogel showed a slight reduction in efficiency, decreasing from 99.05% in the first cycle to 97.04% in the fifth. Meanwhile, CCA-HG_A_ exhibited a more noticeable but still moderate decline, with its efficiency dropping from 96.88% to 92.48%. This difference could be attributed to the compact structure of CCA-HG_A_. However, both hydrogels demonstrated high mechanical strength, strong reusability and maintained effective performance over multiple cycles^13^.

### Proposed mechanism

**Fig. 12:**
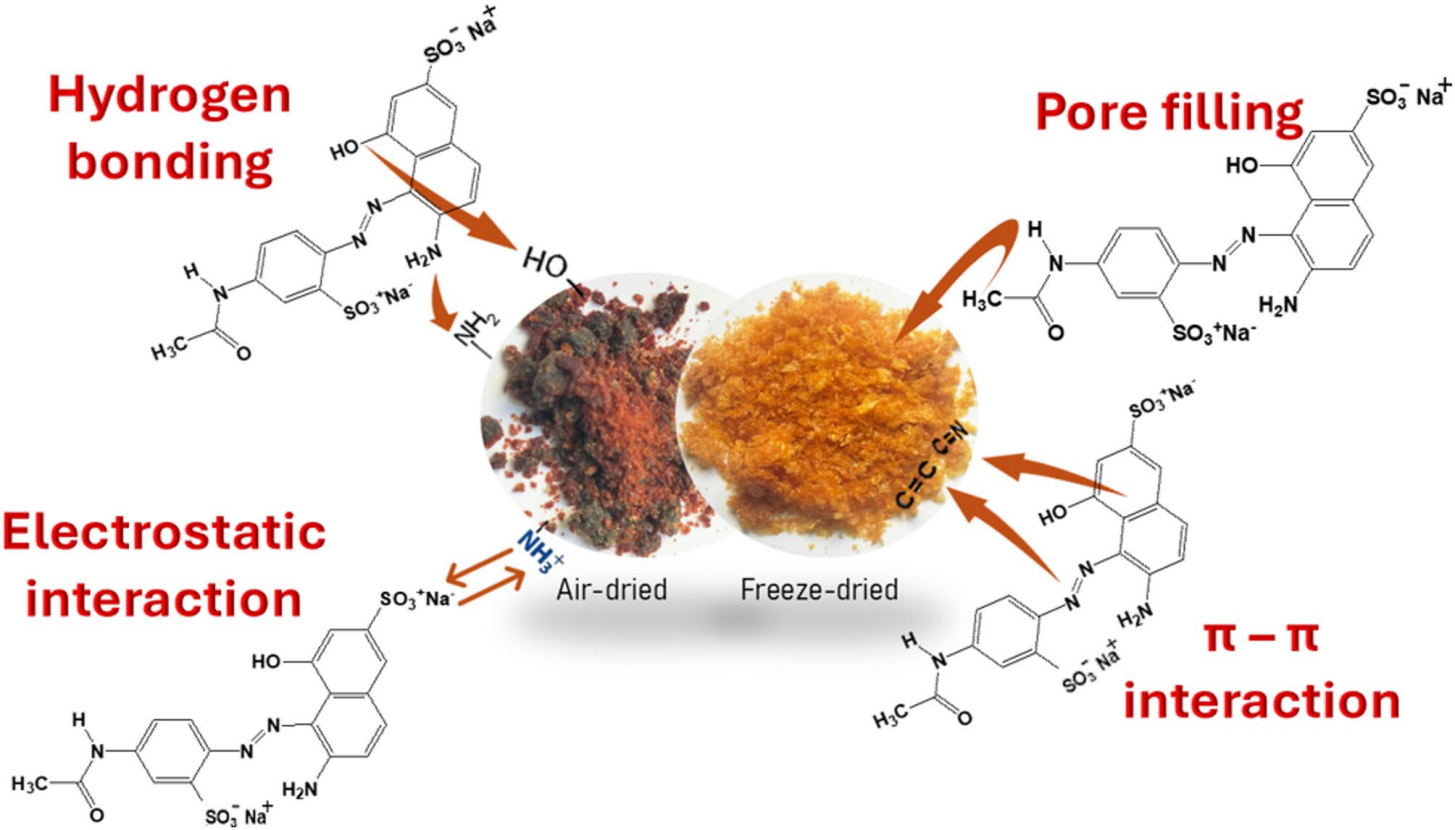
Proposed mechanism of adsorption of AR-37 onto CCA-HG_F_ and CCA-HG_A_.

The proposed mechanism for the adsorption of Acid Red 37 (AR-37) onto the synthesized hydrogels (CCA-HG_F_ and CCA-HG_A_), as shown in Fig. 12, comes out from a proper analysis of adsorption behavior, full characterization before and after the adsorption process, as well as isotherm and kinetics modeling. These approaches offer insights into the probable interactions that occur within the adsorption process. Starting with FTIR analysis, Fig. 1, significant shifts observed in most of the bands after the adsorption confirm that interactions occur between the dye and the hydrogel. These interactions appear to involve the formation of hydrogen bonds between functional groups on the hydrogels (such as –OH and –NH_2_) and the –NH_2_ groups of the dye, emphasizing the role of hydrogen bonding as a primary interaction in binding the dye molecules^80^. The highly porous and mesoporous characteristics of both hydrogels, as demonstrated by SEM and BET analyses, facilitate a pore-filling mechanism, allowing AR-37 molecules to be readily accommodated within the hydrogel matrix^3,11^. Moreover, in acidic conditions and below the point of zero charge, the hydrogel surface acquires a positive charge due to the protonation of –NH_2_ groups in chitosan. This positively charged surface fosters electrostatic attraction with the negatively charged –SO_3_^−^ groups on the dye, which further strengthens the overall adsorption process by adding electrostatic interactions as another key factor^30,81^. XPS results reveal the appearance of new S2p peaks after adsorption which further confirms the successful adsorption process and pronounced shifts in the binding energies of unsaturated groups like C = C and C = N. Such shifts imply the interactions between the aromatic rings and azo groups in AR-37 with the hydrogel’s structure, adding another layer of interaction through π-π stacking that enhances dye attachment to the hydrogel surface^10,26^.

In conclusion, the adsorption of AR-37 onto the synthesized hydrogels is supposed to be driven by a combination of hydrogen bonding, electrostatic interactions, pore-filling and π-π interactions. These complementary forces work together to create a strong and effective adsorption mechanism, enabling the efficient removal of AR-37 from aqueous solutions.

### Comparative study

A comparison with different chitosan-based adsorbents applied to various acid dyes revealed that the synthesized chitosan/cellulose acetate hydrogel demonstrated competitive adsorption capability. When considering the cost-effectiveness, simplicity of preparation, and the use of readily available and non-toxic components, the hydrogel surpassed the other adsorbents. Table 6 summarizes these findings by comparing the adsorption capacity and surface area of the hydrogel to those of other adsorbents. This offers hydrogel as a realistic and sustainable option for dye removal from wastewater. Its ability to remove Acid Red 37, as well as its potential use with other acid dyes, highlights its versatility and effectiveness in environmental applications.

**Table 6:** The adsorptive capacities of various chitosan-based adsorbents for acid dyes.

Adsorbent	Adsorbate	Optimum conditions	q_m_ (mg/g)	Removal efficiency (*R*%)	Surface area (m^2^/g)	Reuse cycles	Refs.
Carbon Nanotube/Chitosan Hydrogel	Acid red 73	pH: 5, Dose: 70 mg, Time: 6 h, Temp: 45 °C	101.07	92.23%	-----	6 cycles (88.52%)	^66^
Quaternized chitosan microspheres	Acid red 18	pH: 8.4, Dose: 0.05 g, Time: 120 min, Temp: 20 °C	142.15	94.06%	4.45	5 cycles (> 85%)	^28^
carboxymethyl β-cyclodextrin–nano chitosan–glutaraldehyde	Acid red 37	pH: 6, Dose: 0.5 g/L, Time: 5 min, Temp: 29 °C	332.60	99.67%	2.903	5 cycles (92.4%)	^13^
Air-dried chitosan/cellulose acetate hydrogel (CCA-HG_A_)	Acid red 37	pH: 1, Dose: 1.2 g/L, Time: 90 min, Temp: 25 °C	20.54	98.30%	52.75	5 cycles (92.4%)	This work
Freeze-dried chitosan/cellulose acetate hydrogel (CCA-HG_F_)	Acid red 37	pH: 1, Dose: 0.2 g/L, Time: 70 min, Temp: 25 °C	149.23	99.53%	94.90	5 cycles (97.04%)	This work

### Geometrical analysis

**Fig. 13:**
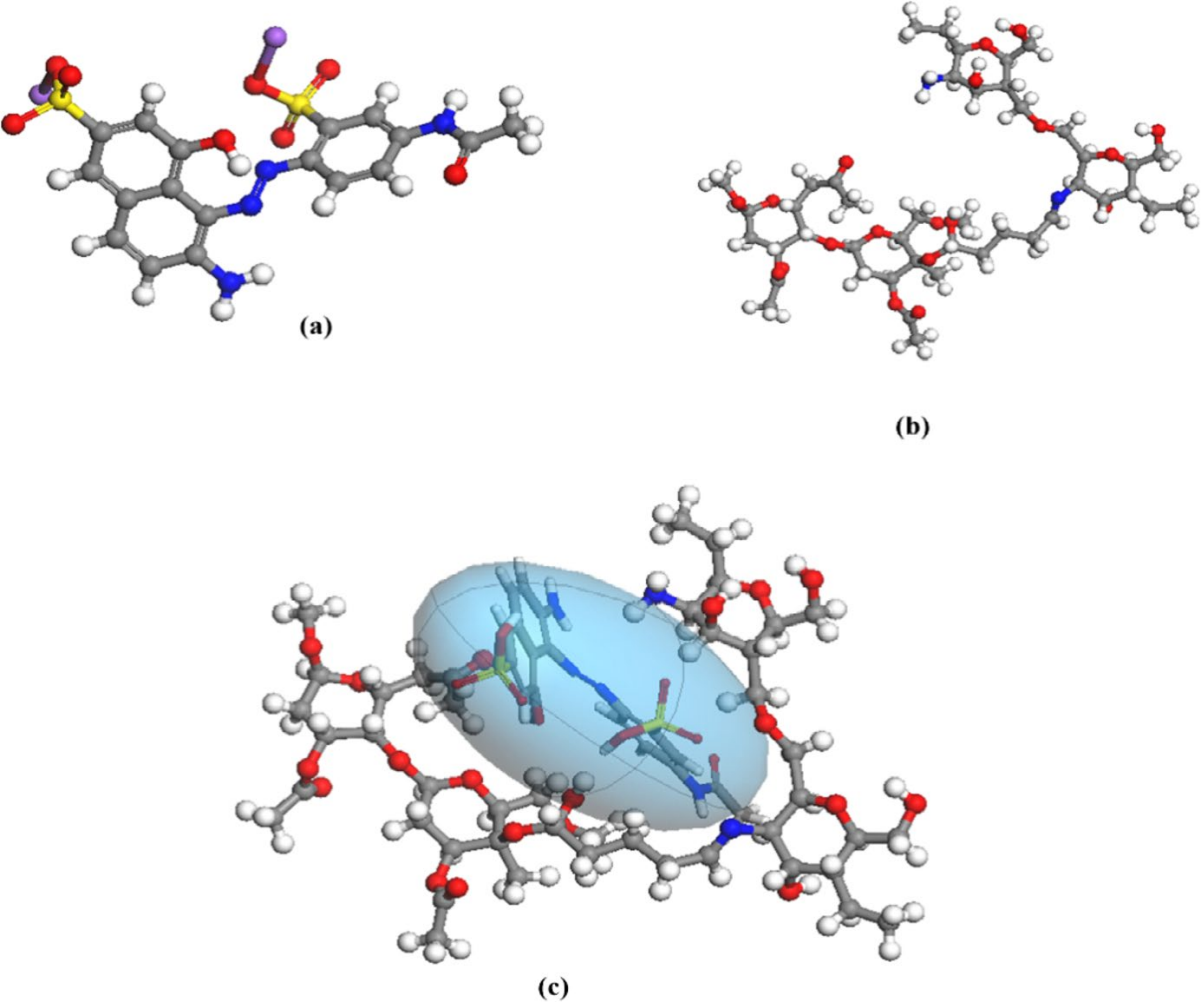
The optimized structures of (**a**) Na-Acid red 37, (**b**) Hydrogel, (**c**) Na-Acid red/Hydrogel with polyhedron adsorbate, using the GGA/PBE method. (C = grey, O = red, N = blue, S = yellow, Na = purple, H= white).

The constructed model represents a biopolymer-based hydrogel composed of crosslinked polysaccharide-like chains with hydrophilic oxygen- and nitrogen-containing groups that allow the material to absorb and retain large amounts of water. The computational model includes two units of chitosan and two units of cellulose acetate crosslinked with a chain arranged in a way that make 3D network. To elucidate the adsorption mechanism, it is essential to characterize the nature of surface interactions between the adsorbent (hydrogel) and the adsorbate (Na-Acid Red 37)^82^. The structural parameters for Na-Acid Red 37 and the hydrogel matrix were optimized using the Dmol³ module within the DFT framework until full convergence was achieved considering water as a solvent in this study. The optimized geometries, obtained using the GGA/PBE functional, are presented in Fig. 13. The optimized geometries of the individual components and the adsorption complex should reveal significant structural features that influence interaction behavior. Na-Acid Red 37 exhibits a planar molecular geometry due to its extended conjugated system, which facilitates π–π stacking interactions with the hydrogel’s aromatic regions. The molecule contains multiple functional groups, such as Na-sulphonate and azo linkages, that serve as active sites for hydrogen bonding and electrostatic interactions. The hydrogel structure, in contrast, shows a semi-flexible polymeric network with varying degrees of porosity and functional moieties (e.g., hydroxyl, carboxyl) exposed on the surface, allowing for multiple binding configurations. Upon adsorption, slight geometric distortions were observed in both Na-Acid Red 37 and the hydrogel, indicating conformational adaptation to maximize interaction. The binding induces local flattening of hydrogel polymer chains near the adsorption site and subtle torsional changes in the dye molecule to align its functional groups with complementary adsorption sites. These geometric adjustments are consistent with strong physisorption behavior supported by non-covalent forces, and they reflect the system’s tendency to achieve a more thermodynamically stable configuration.

#### FMOs analysis

**Fig. 14:**
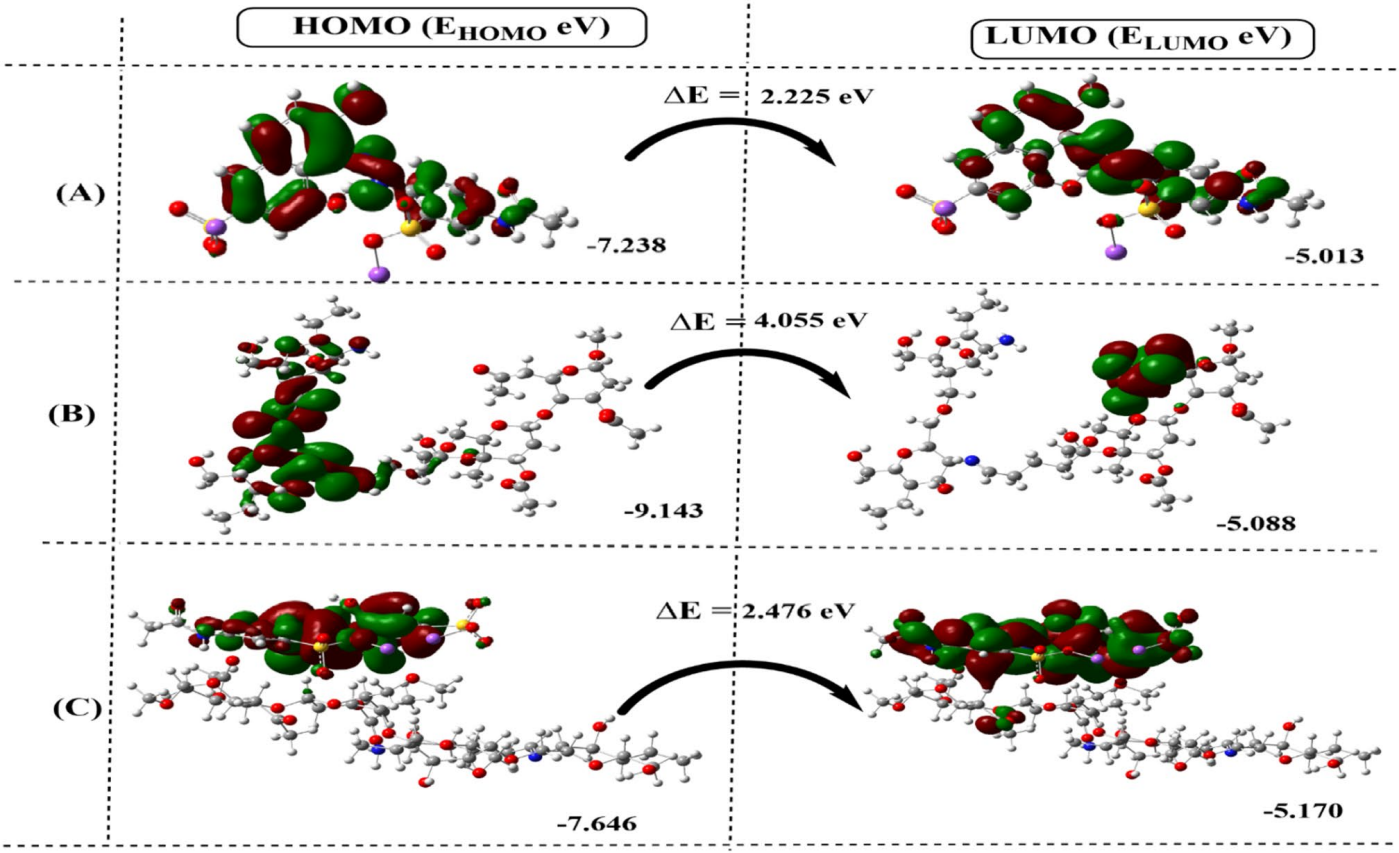
FMOs-transition state and energy values of the designed systems (**A**) Na-Acid red 37, (**B**) Hydrogel and (**C**) Na-Acid red 37/Hydrogel adsorption system.

Considering that Na-Acid Red 37 possesses a smaller energy gap (2.225 eV) compared to the hydrogel matrix (4.055 eV), it can be inferred that the dye molecule reveals a relatively high degree of chemical reactivity towards adsorption. Frontier Molecular Orbital (FMO) analysis of the optimized structures offered valuable insights into the molecular orbital distribution and electronic interactions within the system. As illustrated in Fig. 14, the highest occupied molecular orbital (HOMO) and lowest unoccupied molecular orbital (LUMO) levels were evaluated, revealing the spatial and energetic characteristics of electronic transitions. Upon adsorption, the energy gap between the HOMO and LUMO levels of the Na-Acid Red 37/hydrogel system was reduced to 2.476 eV compared with the unadsorbed surface, indicating enhanced electronic transition potential and improved charge transfer characteristics. This suggests the formation of a new electronic configuration in the excited state, with orbital delocalization extending across the entire adsorbed complex.

#### Molecular dynamics simulation

**Fig. 15:**
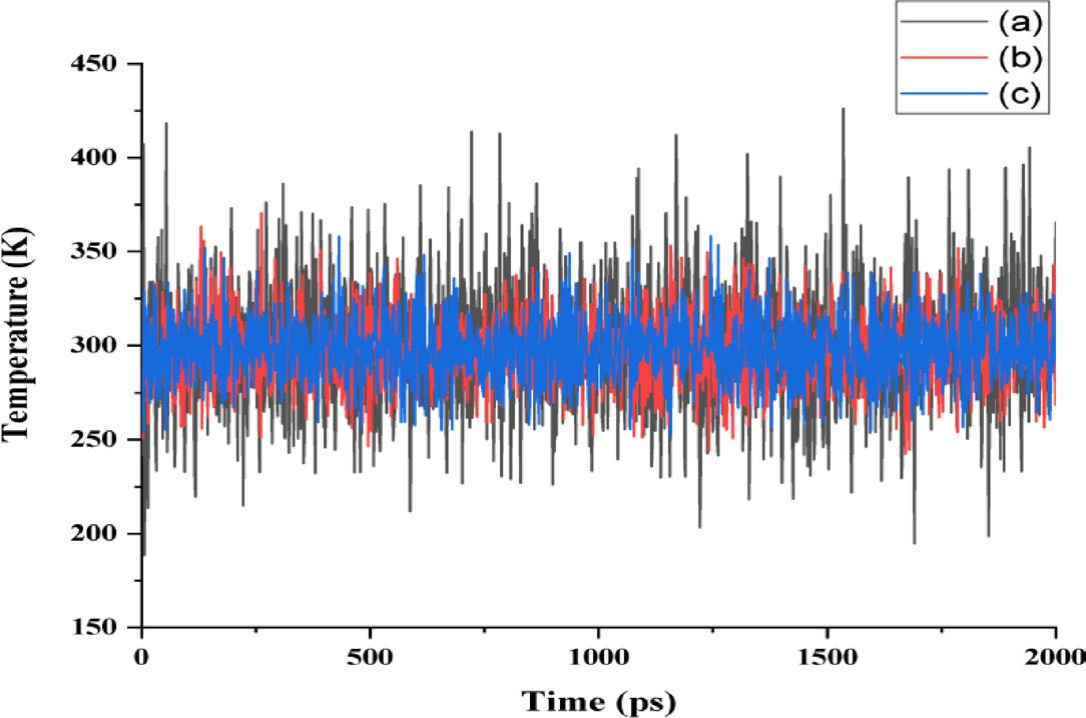
Molecular dynamic simulation of (**a**) Na-Acid red, (**b**) Hydrogel, (**c**) Na-Acid red/Hydrogel through the simulation time.

Molecular dynamics (MD) simulation is a powerful computational technique for investigating the time-dependent motion of atoms and molecules. When applied to polymeric and soft-material systems like hydrogels, along with interacting species such as Na-Acid Red 37, MD simulations can provide valuable insights into the structural flexibility, dynamic behavior, and interaction stability of adsorption systems^83^. In the current study, MD simulations were used to evaluate both the standalone Na-Acid Red 37 and hydrogel systems, as well as their combined adsorption complex. As illustrated in Fig. 15, temperature-controlled simulations revealed similar dynamic responses across all systems. Notably, system stabilization was achieved around 300 K, with initial simulations at maximum temperature of the systems (above 425 K) showing increased atomic fluctuations, which gradually diminished over time, indicating successful equilibration by the end of the simulation process. Figures 2S and 3S display the evolution of energy parameters, specifically potential (E_pot_) and kinetic energy (E_kin_), throughout the MD simulations. The Na-Acid Red 37/hydrogel adsorption system exhibited a higher potential energy profile (550 Kcal/mol) compared to the individual, non-interacting components, suggesting stronger physical interactions and more energetically favorable adsorption. This enhanced potential energy can be attributed to increased atomic relaxation and non-covalent interactions at the hydrogel surface. Additionally, the kinetic energy of the adsorption system (average 170 Kcal/mol) was slightly higher during the early simulation stages, reflecting more dynamic movement and transient flocculation of Na-Acid Red 37 molecules onto the hydrogel network.

#### Adsorption annealing locator

**Fig. 16:**
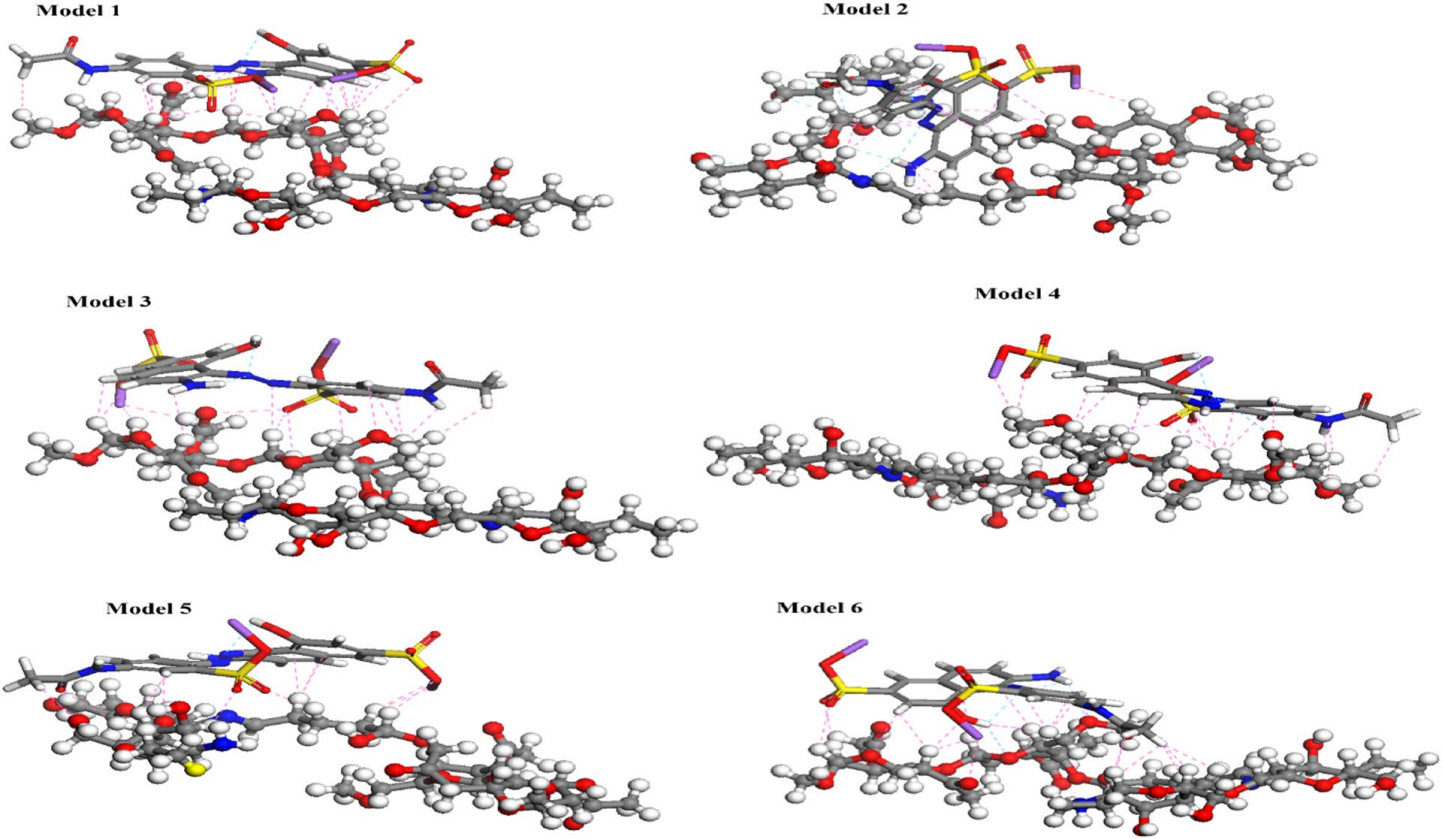
Modules of Molecular adsorption originated from the locator annealing calculation of Na-Acid red/Hydrogel system.

**Fig. 17:**
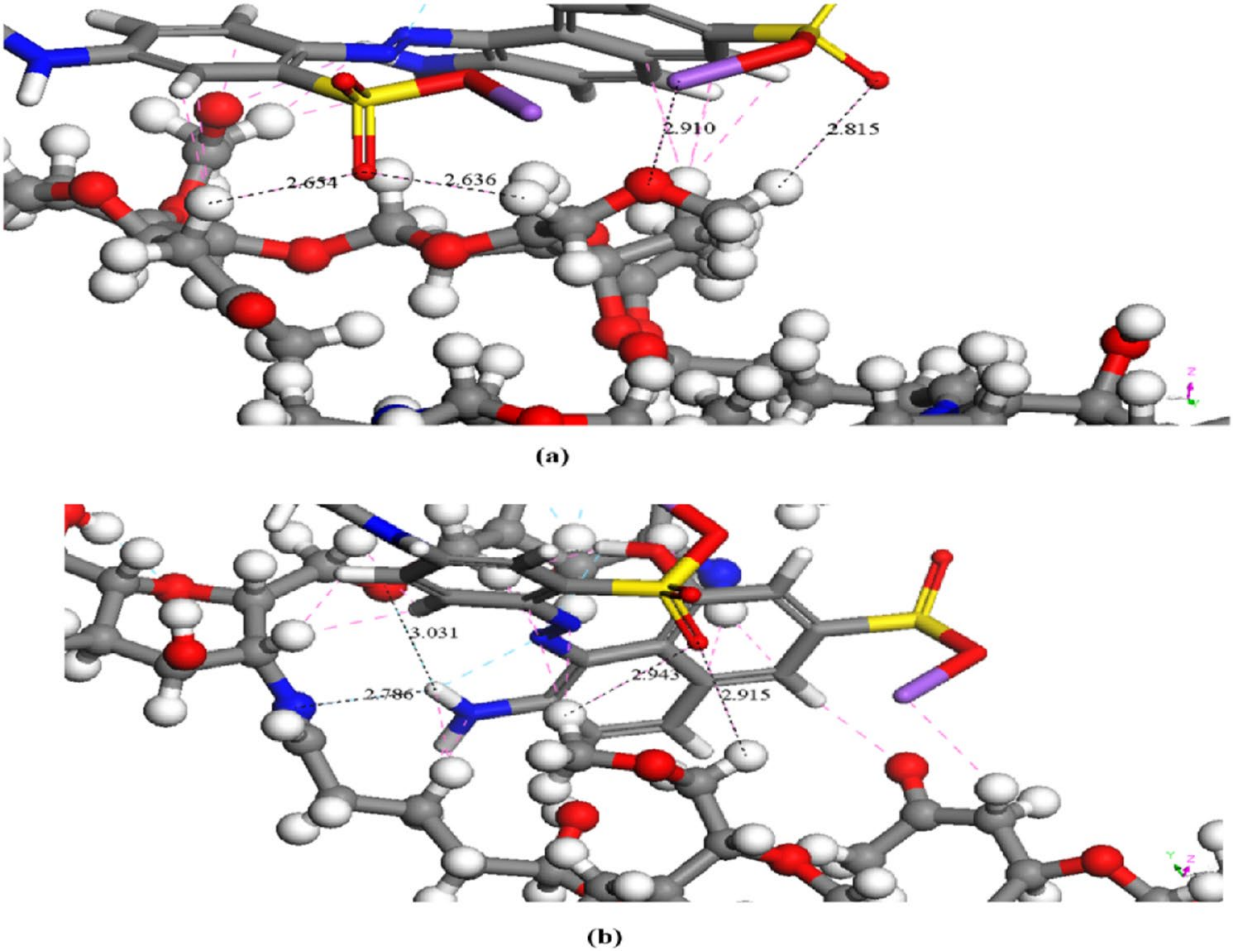
Zoom profile showing the bond distance of several interactions of Na-Acid Red on the surface of the Hydrogel system (**a**) predicted model 1, and (**b**) predicted model 2.

From a structural standpoint, the heteroatoms present in both Na-Acid Red 37 and the hydrogel matrix serve as the primary functional groups that govern surface interactions in the adsorption process^84^. Factors such as the nature of the hydrogel precursor materials, synthesis duration, and preparation conditions can significantly influence the adsorption efficiency and interfacial behavior. The hydrogel system in this study exhibits structural similarities to modified polymeric frameworks incorporating six-membered ring systems functionalized with various polar groups, such as hydroxyl, carboxyl, and sulfonate moieties, that are key contributors to adsorption energy (E_ads_). As illustrated in Fig. 16, the adsorption models demonstrate strong close-range interactions, particularly electrostatic attractions between the hydrogen atoms of the hydrogel and the oxygen or sulfonate functionalities of Na-Acid Red 37. The donor–acceptor relationship becomes apparent when observing the orientation of electron-rich sites on both species, with the hydrogel’s unmodified polar groups often aligning toward the dye molecule. In this context, hydrogen atoms, particularly those adjacent to polar functional groups, act as electron acceptors and participate in H–π donor interactions with the π-electron systems of Na-Acid Red 37. These interactions are further validated by the calculated adsorption energies across several simulated configurations. The most stable adsorption model, Model 1, exhibited an E_ads_ of −24.544 Kcal/mol, while the least favorable model, Model 6, also shows a significant form of interaction with the value of −22.312 Kcal/mol. Variations in adsorption energy can be attributed to differences in conformational alignment and the degree of atomic relaxation within the adsorption complex, highlighting the importance of spatial orientation and surface compatibility in optimizing dye–hydrogel interactions. Further describing the adsorption system, Fig. 17 represents two optimized adsorption configurations of the Na-Acid Red 37/hydrogel system, illustrating the nature and strength of non-covalent interactions at the interface. In Fig. 17a, multiple hydrogen bonding interactions are observed between the sulfonate and carbonyl oxygen atoms of the Na-Acid Red 37 dye and the hydroxyl groups of the hydrogel network. The interatomic distances (2.636 Å and 2.654 Å) indicate strong hydrogen bonding, while additional electrostatic interactions are present between sodium ions and electron-rich oxygen centers (2.910 Å and 2.815 Å). These close contacts suggest significant adsorption stabilization via both hydrogen bonding and ion–dipole interactions. In Fig. 17b, a different adsorption configuration is shown, where π–π stacking between the aromatic ring of Na-Acid Red 37 and aromatic domains within the hydrogel polymer is complemented by multiple hydrogen bonding interactions. The interaction distances (e.g., 2.786 Å, 2.943 Å, 2.915 Å, and 3.031 Å) confirm moderate to strong non-covalent binding, with nitrogen-containing functional groups contributing to hydrogen donor–acceptor interactions. This configuration highlights the importance of spatial alignment in enhancing dye–polymer compatibility and electron delocalization across the adsorption interface. Overall, both models demonstrate that the adsorption of Na-Acid Red 37 onto the hydrogel surface is driven by a combination of hydrogen bonding, electrostatic interactions, and π–π stacking, all of which contribute to the formation of a stable hybrid system with optimized binding orientations.

## Conclusion

Chitosan/cellulose acetate hydrogels were synthesized via glutaraldehyde crosslinking, with a comprehensive comparative analysis of freeze-dried (CCA-HG_F_) and air-dried (CCA-HG_A_) forms. The novelty of this work lies in the uncommon use of cellulose acetate as a hydrogel component (compared to more conventional cellulose derivatives) combined with a direct morphological and performance comparison of the two drying methods. Incorporating cellulose acetate enhanced the structural and functional properties of the hydrogels beyond conventional systems, highlighting their promising potential for advanced adsorption applications. BET analysis revealed that CCA-HG_F_ possessed a significantly larger surface area (94.9 m²/g) and smaller pore diameter (3.12 nm) than CCA-HG_A_ (52.7 m²/g, 3.64 nm), resulting in superior adsorption performance. Both hydrogels exhibited high removal efficiencies toward Acid Red 37 (AR-37) dye, with maximum adsorption capacities of 149.30 mg/g (CCA-HG_F_) and 20.50 mg/g (CCA-HG_A_), respectively. The optimal removal was achieved under acidic conditions, and adsorption equilibrium was reached within a short contact time. Although the maximum adsorption occurs at pH 1, the hydrogel retains > 85% removal efficiency in the pH range of 4–7, confirming its practical utility for treating textile wastewater without extreme acidification. The adsorption data were best described by the pseudo-second-order kinetic model, while nonlinear fitting also showed that the pseudo-first-order model provided an excellent description, with q_e_ values very close to the experimental ones, indicating contributions from both chemisorption and physisorption. Isotherm studies confirmed Langmuir behavior for both hydrogels, with nonlinear fitting providing slightly refined parameters compared to the linear analysis, supporting the reliability of the isotherm parameters and the coexistence of monolayer and multilayer adsorption. The adsorption process was exothermic and spontaneous, and both hydrogels retained excellent reusability across multiple cycles. Preliminary trials with other anionic dyes further demonstrated promising removal efficiency, suggesting the potential versatility of the synthesized hydrogels. Complementary computational modeling confirmed the molecular-level mechanism of adsorption. DFT calculations revealed a significant decrease in the HOMO–LUMO energy gap (ΔE = 2.476 eV) upon dye adsorption, indicating strong electronic interaction and favorable charge transfer. Molecular dynamics simulations confirmed the structural stability of the hydrogel–dye complex under ambient conditions, with dye stabilization inside the hydrogel network. Adsorption energy values (–24.544 to − 22.312 Kcal/mol) supported the spontaneous nature of the process, highlighting the synergistic contributions of hydrogen bonding, π–π stacking, electrostatic attraction, and pore-filling. However, the novelty in this work is based on the specific combination of a 3D chitosan/cellulose acetate hydrogel crosslinked with minimal glutaraldehyde (demonstrating that cellulose acetate, typically limited to membranes, can be engineered into a high-performance 3D hydrogel, unlocking its potential in water treatment applications), with a systematic comparison of freeze-drying and air-drying effects, comprehensive characterization, and integration of experimental and computational adsorption studies. Furthermore, the high adsorption capacity, particularly of the freeze-dried (CCA-HG_F_), means that a small mass of adsorbent can treat a large volume of contaminated water, amplifying the functional benefit derived from the minimal chemical input. Future work will explore fully bio-based crosslinkers (e.g., genipin, tannic acid). However, the present study successfully establishes that even with a minimized amount of a conventional crosslinker, the net environmental benefit over enhanced performance, reusability, and the use of abundant biopolymer feedstocks (chitosan and cellulose acetate) establishes these hydrogels as a significant step toward more sustainable water treatment technologies.

## Supplementary Information

Below is the link to the electronic supplementary material.

Supplementary Material 1

## Data Availability

Data will be made available on request.
