# Supplementary material for: Synthesis of a 3D chitosan/cellulose acetate hydrogel: comparative impact of drying techniques on morphology for sustainable wastewater treatment
Source: Sci Rep. 2026 Jun 8;16:17674. doi: 10.1038/s41598-026-53193-0 (PMC13246751; doi:10.1038/s41598-026-53193-0)
Supplement: Supplementary file 1 — Supplementary Material 1 [file 41598_2026_53193_MOESM1_ESM.docx]

**Supplemental Materials**

Synthesis of a 3D Chitosan/Cellulose Acetate Hydrogel: Comparative Impact of Drying Techniques on Morphology for Sustainable Wastewater Treatment

Laila M. boghdady^[[1]](#footnote-1)^*^1^, Mekhamer Wafaa Kotb^2^, Hemmat A. Elbadawy^[[2]](#footnote-2)^, Doaa S. El-Sayed^1^, Amel F. Elhussieny^1^, Ali El-Dissouky^1^

^1^Chemistry Department, Faculty of Science, Alexandria University, Alexandria, Egypt, ^2^ Department of Material Science, Institute of Graduate Studies & Research, Alexandria University, Alexandria, Egypt.

# **Materials and methods**

# **1. Materials**

Chitosan (degree of deacetylation ≈ 75% and low molecular weight (50.000-190.000) )was purchased from Alpha Chemika, cellulose acetate (extra pure with assay acetyl group = 28-45%) was purchased from Laxness’ Energizing Chemistry), glutaraldehyde was purchased from advent Chembio Pvt ltd, acetic acid glacial and acetic acid (96%) were purchased from Gateway, absolute ethanol was purchased from Sigma Aldrich Chemical Company, Germany, sodium chloride was purchased from MP Biomedicals and acid red 37 (C_18_H_14_N_4_Na_2_O_8_S_2_, 524.44 g/mol) was purchased from Chemajet.

# **2. Instrumentation**

The concentration of the AR-37 was determined using a double beam UV–Vis spectrophotometer (T60 + UV/Vis spectrometer, PG Instruments Ltd., UK) at λ = 504 nm at central laboratory unit, Faculty of Science, Alexandria University. pH meter (HANNA Instruments pH -211 Microprocessor pH-meter) was used for measurement of pH. The function groups of the synthesized hydrogels were determined by Fourier transform infrared spectroscopy (FTIR) (Tensor 37, Bruker, Germany) in the range 400–4000 cm^− 1^ at the Central Laboratory, Faculty of Science, Alexandria University. The crystallinity and the phase of the synthesized hydrogel species were analyzed by powder X-ray Diffraction (XRD) analysis with XRD-D2 phaser, Bruker, Germany) using Cu Kα radiation (λ = 1.54 Å) over 2θ range of 5–60^◦^ and scan rate of 2^◦^/min at the Central Laboratory, Faculty of Science, Alexandria University. The morphology of both hydrogels was evaluated by Scanning Electron Microscopy (SEM) using a JEOL-SEM operating at an accelerating voltage of 20.00 kV equipped at the Electron Microscope Unit, Faculty of Science - Alexandria University. The chemical composition of the sample was investigated by the X-ray photoelectron spectroscopy (XPS) at Central Metallurgical Research and Development Institute with monochromatic X-ray Al K-alpha radiation − 10 to 1350 eV spot size 400 μm at pressure 10–9 mbar with full spectrum energy 200 eV and at narrow spectrum 50 eV. Brunauer-Emmett-Teller (BET) was used to measure the specific surface area and pore size distribution of the fabricated hydrogels via N_2_ adsorption/desorption isotherms under 77.4 K and a 0.001–1 relative pressure (P/P°) range using BELSORP-Mini II, BEL Japan, Inc analyzer at the National institute of oceanography and fisheries (NIOF).

## **Batch method for adsorption of AR-37**

A Stock solution of AR-37 (250.0 mg/L) was prepared by dissolving 0.250 g of the dye in one-liter distilled water. The efficiency of the synthesized hydrogel for the removal of AR-37 was investigated by batch adsorption experiments as a function of pH, dose of the adsorbents, initial concentration of the adsorbate, contact time and temperature. Each batch adsorption experiment was performed in stoppered conical flasks containing a 25.0 mL of AR-37 solution with different concentrations (varying from 5.0 to 50.0 mg/L) and different masses of the synthesized hydrogels (varying from 0.001 to 0.040 g) at different pH (1-10). The mixtures were shaken using orbital shaker incubator at 100 rpm for different time periods (ranging from 5 to 120 minutes) at room temperature (25^o^C). Each batch adsorption experiment was conducted in triplicate to ensure result accuracy.

Removal percentage of AR-37 (R%) and adsorption capacity (q mg/g) were evaluated by using equations **1** and **2** respectively [1].

$\boldsymbol{\%R=}\frac{\boldsymbol{C}_{\boldsymbol{0}}\boldsymbol{-}\boldsymbol{C}_{\boldsymbol{e}}}{\boldsymbol{C}_{\boldsymbol{0}}}\boldsymbol{\times100}$ **1**

$\boldsymbol{q=}\frac{\boldsymbol{(}\boldsymbol{C}_{\boldsymbol{0}}\boldsymbol{-}\boldsymbol{C}_{\boldsymbol{e}}\boldsymbol{)\times V}}{\boldsymbol{m}}$ **2**

where, C_o_ and C_e_ are the initial and final concentrations (mg/L) of AR-37, respectively, V is the volume of dye solution (L), and m is the mass of adsorbent (g).

- - 1. **Effect of pH**

The pH (1–10) of the AR-37 solution was adjusted using HCl (0.10 M) or NaOH (0.10 M) solutions. Adsorbent doses of 0.2 g/L (CCA-HG_F_) and 0.4 g/L (CCA-HG_A_) were used by adding the appropriate mass of hydrogel to 25.0 mL of AR-37 solution (25.0 mg/L), and the mixtures were shaken at 100 rpm for 30 minutes at room temperature (25 °C).

- - 1. **Effect of adsorbent dosage**

Various adsorbent doses were tested by adding different amounts of CCA-HG_F_ and CCA-HG_A_ to 25.0 mL of AR-37 solution (initial concentration 25.0 mg/L) at the optimum pH (pH 1 for both hydrogels). The applied dose ranged from 0.04 to 0.4 g/L for CCA-HG_F_ and from 0.08 to 1.6 g/L for CCA-HG_A_. The mixtures were shaken at room temperature (25 °C) for 30 minutes

- - 1. **Effect of initial concentration**

The optimum doses (0.2 g/L for CCA-HGF and 1.2 g/L for CCA-HGA) were added to 25 mL of AR-37 solutions with various initial concentrations (5.0–50.0 mg/L) at the optimum pH and shaken for 30 minutes at room temperature.

- - 1. **Effect of contact time**

Adsorbent doses of 0.2 g/L (CCA-HGF) and 1.2 g/L (CCA-HGA) were added to 25.0 mL of AR-37 solutions with initial concentrations of 30.0 mg/L and 25.0 mg/L, respectively, at the optimum pH. The mixtures were then shaken at room temperature for different time intervals ranging from 5 to 120 minutes to investigate the effect of contact time.

- - 1. **Effect of temperature**

Under optimum conditions, 0.2 g/L of CCA-HGF and 1.2 g/L of CCA-HGA were added to 25.0 mL of AR-37 solutions with initial concentrations of 30.0 mg/L and 25.0 mg/L, respectively. The mixtures were shaken for 75 and 90 minutes, respectively, at various temperatures (25, 30, 35, and 40 °C).

- - 1. **Point of zero charge (pH_PZC_)**

A dose of 0.4 g/L of both CCA-HGF and CCA-HGA was added to 25.0 mL of 0.10 M NaCl solutions at different initial pH values (pHo) ranging from 1 to 10, adjusted using 0.10 M HCl or NaOH in stoppered bottles. The suspensions were shaken for 24 hours, after which the final pH (pHf) of each solution was measured. The point of zero charge (PZC) was determined as the intersection point of the curve plotted between pHf and pHo[2].

- - 1. **Effect of foreign ion**

Under the optimized conditions, each adsorbent was added to a series of 100 mL reagent bottles containing 25.0 mL of AR-37 solution. To assess the influence of various anions on the adsorption performance, sodium salts (NaCl, NaNO₃, Na₂SO₄, and Na₃PO₄) were added to achieve a final concentration of 0.01 M. The mixtures were shaken, and the removal efficiencies were subsequently calculated [3] .

**Tables**

**Table S1. Equations describing adsorption isotherm models**[1]**.**

| Isotherm model | linear form | nonlinear form | parameters |
| --- | --- | --- | --- |
| Langmuir | $\frac{c_{e}}{q_{e}}=\frac{1}{q_{m}K_{L}}+\frac{c_{e}}{q_{m}}$  $R_{L}=\frac{1}{1+K_{L}c_{0}}$ | qe = qm K_l_Ce/ 1 + K_l_C_e_ | C_e_ equilibrium concentration in solution (mg L^−1^) |
|  |  |  | q_e_ amount of adsorption at equilibrium (mg/g) |
|  |  |  | q_m_ maximum adsorption capacity (mg/g) |
|  |  |  | K_L_ Langmuir isotherm constant (L/mg) |
|  |  |  | R_L_ separation factor (0 < R_L_ < 1) |
|  |  |  | C_o_ initial adsorbate concentration (mg L^−1^ ). |
| Freundlich | log q_e_ = log K_f_ + $\frac{1}{n}$ (log C_e_) | qe = K_f_ Ce^1/n^ | K_f_ measure of adsorption capacity (mg/g) |
|  |  |  | 1/n adsorption intensity |
| Temkin | q_e_ = $\frac{R_{T}}{b_{Te}}$ lnK_Te_ +$\frac{R_{T}}{b_{Te}}$ lnC_e_ | qe =( RT/ b) lnKT_e_C_e_ | K_Te_ Temkin isotherm constant (L/g) |
|  |  |  | b_Te_ Temkin constant related to heat of sorption (J mol^−1^), R gas constant |
|  |  |  | T absolute temperature (K) |
| Dubinin-Radushkevich | ln q_e_ = ln q_m_ – B ε^2^  ε = RT ln [1+ $\frac{1}{c_{e}}$]  E = 1/(2B)^0.5^ | qe = q_m_ e^−B^ ^ε2^ | ε Polanyi potential |
|  |  |  | E mean free energy of sorption (KJ mol^−1^ ). |
|  |  |  | θ the degree of surface coverage |
| Flory-Huggins | log $\frac{\theta}{c_{o}}$ = log k_FH_ + n_FH_ log (1-θ)  θ =$\frac{1-c_{e}}{c_{o}}$  K_FH_ = exp. ($-\frac{G_{o}}{RT}$) |  | k_FH_ the Flory-Huggins equilibrium constant, |
|  |  |  | n_FH_ model exponent |
|  |  |  | ΔG_o_ the standard free energy change |

**Table S2. Equations describing adsorption Kinetic models**[2]**.**

| kinetic model | Linear Equation | Nonlinear equation | Parameters |
| --- | --- | --- | --- |
| pseudo-first order | Ln (q_e_-q_t_) = ln q_e_ -k_1_t | qt = qe(1 − e^− K1 t^ ) | 𝑞_𝑒_ (mg g^-1^); amount of dye adsorbed on adsorbent at equilibrium |
|  |  |  | 𝑞_𝑡_ (mg g^-1^) amount of dye adsorbed on adsorbent at time t |
|  |  |  | t; time (s) |
|  |  |  | 𝐾_1_ (s^-1^) is the rate constant |
| pseudo-second order | $\frac{t}{q_{t}}=\frac{1}{k_{2}q_{e}^{2}}+\frac{1}{q_{e}}t$ | qt = K_2_q _e_^2^ t /1 + K_2_qe t | 𝐾_2_ (g mg^-1^ s ^-1^) is the rate constant of the pseudo-second order |
| Elovich | $q_{t}=\frac{1}{\beta}\ln\alpha\beta+\frac{1}{\beta}\ln t$ | qt = (1/β)ln(αβt + 1) | α; the initial adsorption rate (mg g^-1^ s ^-1^) |
|  |  |  | β; the surface coverage and activated energy (g/mg). |
| Intra-particle diffusion mode | $q_{t}=k_{ip}\times t^{0.5}+c_{i}$ | qt = k_id_*t^0.5^ + C_i_ | k_id_ (mg g^-1^ s ^-1/2^) is the intraparticle diffusion rate constant |
|  |  |  | C_i_ is the external film resistance which indicates the thickness of boundary layer |

**Table S3. Thermodynamic parameters relations**[3]**.**

| Equation | Parameters |
| --- | --- |
| $\boldsymbol{ln}\boldsymbol{K}_{\boldsymbol{d}}\boldsymbol{=-}\left( \frac{{\boldsymbol{\Delta}\boldsymbol{H}}^{\boldsymbol{o}}}{\boldsymbol{RT}}\boldsymbol{+}\frac{\boldsymbol{\Delta}\boldsymbol{S}^{\boldsymbol{o}}}{\boldsymbol{R}} \right)$  ΔG^o^ = -R T ln K_d_  ΔG^o^ = ΔH^o^-T ΔS^o^  $\boldsymbol{K}_{\boldsymbol{d}}\boldsymbol{=-}\frac{\boldsymbol{c}_{\boldsymbol{e}}}{\boldsymbol{c}_{\boldsymbol{o}}}$ | R; the universal gas constant (8.314 J mol^-1^K ^-1^) |
|  | T; the absolute temperature (°K) |
|  | K_d_; the distribution constant (dimensionless) |
|  | C_o_; the original concentration (mg L^-1^) |
|  | C_e_; the equilibrium concentrations (mg L^-1^) |
|  | ΔG^o^, free energy change (kJ/mole) |
|  | ΔH^o^, enthalpy change (kJ/mole) |
|  | ΔS^o^, entropy change (kJ/mole/^o^K) |

**Table S4 .**Comparative analysis of key adsorption parameters for AR-37 onto CCA-HGF and CCA-HGA with other chitosan-based adsorbents from the literature.

| Adsorbent | **Adsorbate** | **Isotherm Parameters (Langmuir)** | **Kinetic Parameters (Pseudo-2nd Order)** | **Thermodynamic Parameters** | **Reference** |
| --- | --- | --- | --- | --- | --- |
| CCA-HG_F_ | AR-37 | q_max_ (mg/g): 175.44  R^2^: 0.99 K_L_ (L/mg): 0.75 | k_2_ (g/mg.min): 0.001  q_e_ (mg/g): 161.29  R^2^: 1 | ΔG (kJ/mol):-17.95  ΔH (kJ/mol): -162.83  ΔS (J/mol.K): -0.49 | Current Study |
| CCA-HG_A_ | AR-37 | q_max_: 38.46 mg/g R^2^: 0.98  K_L_ (L/mg): 0.39 | k_2_ (g/mg.min): 0.005  q_e_ (mg/g): 22.173  R^2^: 1 | ΔG (kJ/mol): -9.36  ΔH (kJ/mol): -170.89  ΔS (J/mol.K): -0.54 | Current Study |
| Chitosan/Squid Pen Protein | RB4 | qmax: 151.52 mg/g  R^2^: 0.985 K_L_ (L/mg): 0.187 | k_2_: 7.85 x 10^−4^ g/mg.min  q_e_: 110 mg/g  R^2^: >0.99 | Not specified | [4] |
| Chitosan-Montmorillonite | Reactive Red 120 | q_max_: 500 mg/g  R^2^: 0.999 K_L_ (L/mg): 0.027 | k_2_: 1.25 x 10^−3^ g/mg.min  q_e_: 125 mg/g  R2: 0.998 | ΔG: -21.45 kJ/mol  ΔH: -35.2 kJ/mol  ΔS: 46.2 J/mol.K | [5] |
| Chitosan-Alginate | Reactive Red X-3B | q_max_: 1834.04 mg/g  R^2^: >0.99 K_L_ (L/mg): 0.003 | k_2_: 8.9 x 10^−5^ g/mg.min  q_e_: 1834 mg/g  R^2^: >0.99 | ΔG: -8.97 kJ/mol  ΔH: 22.4 kJ/mol  ΔS: 96.5 J/mol.K | [6] |
| Chitosan-Graphene Oxide | Acid Orange 10 | q_max_: 81 mg/g  R^2^: 0.992 K_L_ (L/mg): 0.02 | k_2_: 2.09 x10^−3^ g/mg.min  q_e_: 70 mg/g  R^2^: 0.995 | ΔG: -10.4 kJ/mol  ΔH: -15.8 kJ/mol  ΔS: -17.9 J/mol.K | [7] |

**Figures**

**Fig. S1. X-ray diffraction (XRD) pattern of CCA-HG_F_ and CCA-HG_A_.**


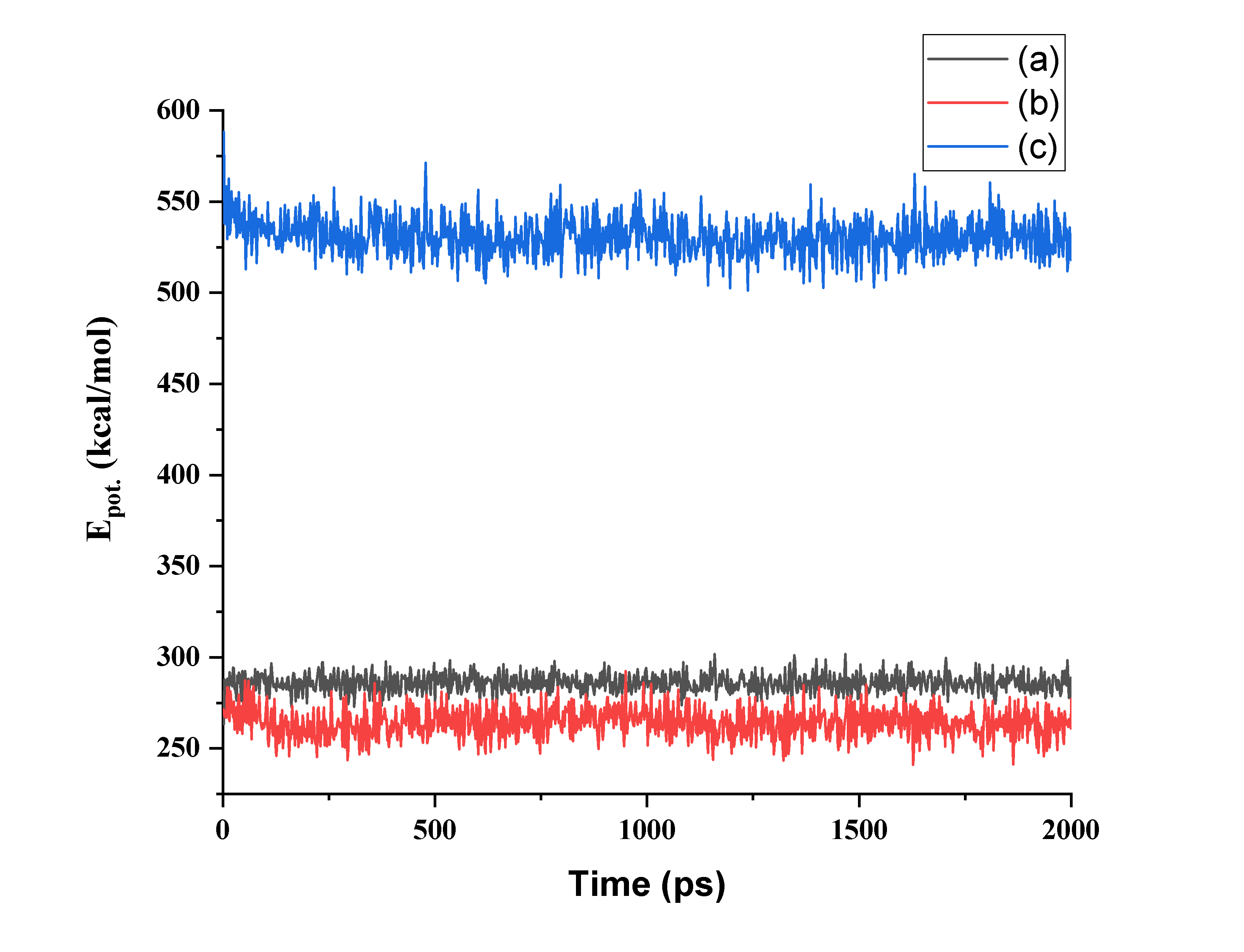


**Fig. S2. Potential energy during dynamic simulation of (a) Na-Acid red, (b) Hydrogel, (c) Na-Acid red/Hydrogel.**


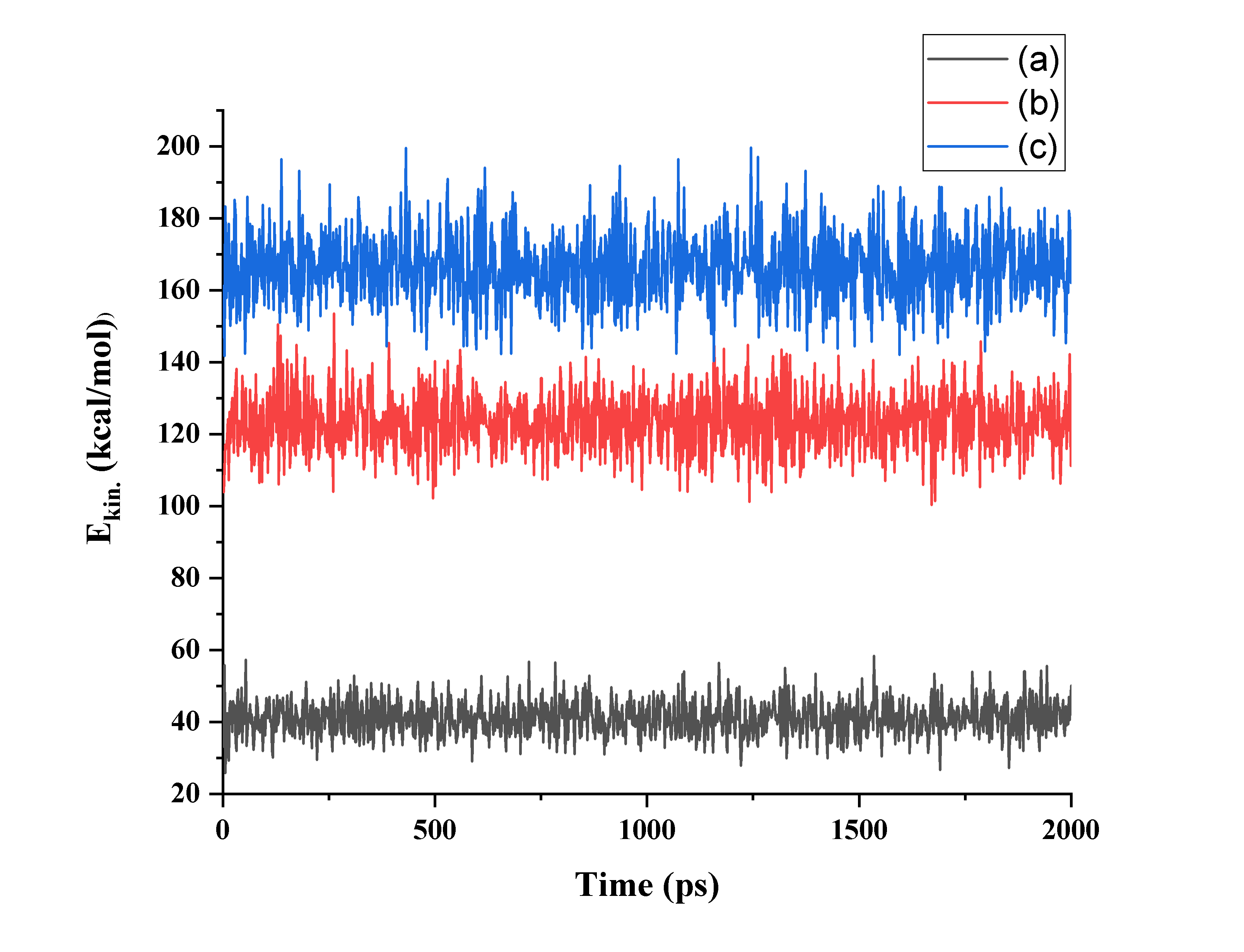


**Fig. S3. Kinetic energy during dynamic simulation of (a) Na-Acid red, (b) Hydrogel, (c) Na-Acid red/Hydrogel.**

# **References**

[1] T.E. Khalil, A.H. Abdel-Salam, L.A. Mohamed, E. El-Meligy, A. El-Dissouky, Crosslinked modified chitosan biopolymer for enhanced removal of toxic Cr(VI) from aqueous solution, Int J Biol Macromol 234 (2023). <https://doi.org/10.1016/j.ijbiomac.2023.123719>.

[2] M.M. Emara, S.T. Hafez, T.E. Khalil, A.E.H.B. Kashyout, A. El-Dissouky, D.S. El-Sayed, Electronic and structural perturbations of microporous ZIF-67 nanoparticles and Cr(VI) molecule during adsorptive water decontamination unveiled by experimental and quantum computational investigations, J Mol Liq 390 (2023). <https://doi.org/10.1016/j.molliq.2023.123042>.

[3] H.A. Elbadawy, A. El-Dissouky, S.M. Hussein, S.R. El-Kewaey, S.A. Elfeky, G. El-Ghannam, A novel terpolymer nanocomposite (carboxymethyl β-cyclodextrin–nano chitosan–glutaraldehyde) for the potential removal of a textile dye acid red 37 from water, Front Chem 11 (2023). <https://doi.org/10.3389/fchem.2023.1115377>.

[4] P.Y.S. Nakasu, M.A. Martinez, S. Melanie, T.A. Shmool, J.P. Hallett, Chitosan-Based Biocomposite Hydrogels with Squid Pen Protein for Anionic Dyes Adsorption, ACS Mater Lett 7 (2025) 1012–1018. <https://doi.org/10.1021/acsmaterialslett.4c01802>.

[5] J. Li, J. Cai, L. Zhong, H. Wang, H. Cheng, Q. Ma, Adsorption of reactive dyes onto chitosan/montmorillonite intercalated composite: multi-response optimization, kinetic, isotherm and thermodynamic study, Water Science and Technology 77 (2018) 2598–2612. <https://doi.org/10.2166/wst.2018.221>.

[6] M.M. ALSamman, J. Sánchez, Chitosan- and Alginate-Based Hydrogels for the Adsorption of Anionic and Cationic Dyes from Water, Polymers (Basel) 14 (2022). <https://doi.org/10.3390/polym14081498>.

[7] Preeti, M. Bhandari, N. Kumari, Adsorption Efficiency and Reusability of Graphene Oxide and Graphene Oxide‐Mt‐Chitosan Composite for Cationic and Anionic Dyes, ChemistrySelect 10 (2025). <https://doi.org/10.1002/slct.202406202>.

1. * Corresponding author e-mail: [laila.boghdady_pg@alexu.edu.eg](mailto:laila.boghdady_pg@alexu.edu.eg), [lailamohamed20166@gmail.com](mailto:lailamohamed20166@gmail.com) [↑](#footnote-ref-1)
2. [↑](#footnote-ref-2)
